# Ketogenic diet as a therapeutic intervention for biochemical hyperandrogenism in PCOS women with obesity or overweight: a meta-analytic study

**DOI:** 10.1007/s40618-026-02924-1

**Published:** 2026-06-13

**Authors:** Elisa Maseroli, Chiara Alfaroli, Michela Cirillo, Serena Anna Ravelli, Irene Lambrinoudaki, Martina Focardi, Gianni Virgili, Linda Vignozzi

**Affiliations:** 1 Andrology and Gender Endocrinology Unit, Careggi University Hospital, Florence, Italy; 2 Department of Experimental and Clinical Biomedical Sciences “Mario Serio”, University of Florence, Florence, Italy; 3 Second Department of Obstetrics and Gynecology, School of Medicine, Aretaieion University Hospital, National and Kapodistrian University of Athens, Athens, Greece; 4 Forensic Medical Sciences, Department of Health Science, University of Florence, Florence, Italy; 5 Department of Neurosciences, Psychology, Drug Research and Child Health (NEUROFARBA), University of Florence, Florence, Italy; 6 I.N.B.B. (Istituto Nazionale Biostrutture E Biosistemi), Rome, Italy

**Keywords:** Polycystic ovary syndrome (PCOS), Ketogenic diet, Hyperandrogenism, Reproductive outcomes, Insulin resistance, Obesity

## Abstract

**Purpose:**

To assess whether the Ketogenic Diet (KD) is associated with improvements in biochemical markers of hyperandrogenism in women diagnosed with Polycystic Ovary Syndrome (PCOS) with obesity or overweight.

**Methods:**

A systematic search was conducted in five databases: MEDLINE (via PubMed), Embase, Cochrane Central Register of Controlled Trials (CENTRAL), Scopus, and Web of Science. Both RCTs and pre-post studies providing data for direct extraction or calculation of mean (SD) changes in the investigated outcomes, published until March 1, 2025, were considered. Protocols included “Very Low” and “Low” Calories KD, and modified KD regimens. The primary outcome was the effect of the KD on total testosterone (TT), Free T (FT) and Sex Hormone Binding Globulin (SHBG) compared to baseline.

**Results:**

The KD was found to be associated with a statistically significant reduction in TT (n = 290, Mean Difference [MD] -0.35, CI -0.46; -0.24) and a significant increase in SHBG levels (n = 144, MD 19.50, CI 6.90; 32.11), independently of study design. When considering only RCTs (4 studies), the KD showed a moderate, statistically significant benefit on TT levels compared to the control intervention, with a high heterogeneity (n = 115 vs. 117, SMD -0.284, CI -0.543, -0.035; p = 0.031). Neither the duration of the KD protocol, the change in BMI, the change in fasting insulin levels nor the change in LH/FSH ratio were able to significantly affect the change in TT levels observed over time. In addition, the KD associated with a significant reduction in LH levels (n = 275, MD -3.48, CI -4.71; -2.26) and in the LH/FSH ratio (n = 275, MD -0.99, CI -1.44; -0.54).

**Conclusion:**

Our meta-analysis indicates a reduced biochemical hyperandrogenism—particularly a reduction in TT—and a reduction in the LH/FSH ratio among women with PCOS following a KD. Our findings also suggest a specific effect of ketosis on ovarian function acting alongside metabolic improvements, but a direct causal link cannot be definitively established. Owing to the low quality and heterogeneous nature of the available studies, larger, high-quality RCTs are essential to consolidate this evidence and determine clinical efficacy.

**Supplementary Information:**

The online version contains supplementary material available at https://doi.org/10.1007/s40618-026-02924-1.

## Introduction

Polycystic Ovary Syndrome (PCOS) is one of the most common endocrine disorders affecting women of reproductive age, with a prevalence of 10–13% [1]. The underlying pathogenic mechanisms and risk factors for PCOS are complex and not yet fully understood, with a highly heterogeneous presentation and a significant long-term risk for cardiovascular morbidity [2]. However, research consistently highlights the significant role of metabolic features in the clinical characteristics and complications of the syndrome. As a result, lifestyle intervention is currently regarded as a first line therapy for PCOS. Specifically, exercise alone, or a combination of diet, exercise, and behavioral strategies, is recommended for all women with PCOS to improve metabolic health, including reducing central adiposity and improving lipid profiles [1, 3]. Weight loss interventions are of particular importance in PCOS women with overweight and obesity, since it is recognized that many patients tend to obtain a greater longitudinal weight gain, and/or face greater challenges with weight management compared to non-PCOS women [4].

Although no conclusive evidence supports the superiority of any one diet over another in terms of anthropometric, metabolic, hormonal, reproductive, or psychological outcomes [5], growing research suggests that calorie-restricted diets and low-energy, low-carbohydrate combination diets may offer advantages over other dietary interventions, particularly in improving clinical manifestations and pregnancy rates in individuals with PCOS and a body mass index (BMI) ≥ 25 kg/m^2^ [6].

Among dietary protocols characterized by low carbohydrate intake, the Ketogenic Diet (KD) is distinguished by its high fat content (> 70% of total caloric intake) while limiting excess protein consumption [7]. This approach promotes the production of ketone bodies—primarily acetoacetate and β-hydroxybutyrate—leading to a state of nutritional ketosis. A specific variation of KD, the very low-calorie ketogenic diet (VLCKD), involves a daily carbohydrate intake of less than 30 g/day (approximately 13% of total energy intake), with relatively higher proportions of fat (~ 44%) and protein (~ 43%), while maintaining a total daily energy intake of less than 800 kcal [7]. Recently, VLCKD has been redefined as Very Low-Energy Ketogenic Therapy (VLEKT) by a panel of Italian experts [8], and it has been proposed as a therapeutic approach for obesity. In 2019, the Italian Society of Endocrinology (SIE) recognized PCOS-associated obesity as one of its indications, albeit with a weak recommendation due to the limited evidence available at the time [9]. Since then, evidence has been accumulating; however, no updated consensus has been issued to strengthen the recommendation.

In this context, studying the effects of nutritional ketosis on hormonal and reproductive parameters—key aspects of PCOS clinical presentation—is particularly important. This research is essential for providing healthcare providers with updated evidence-based data to guide nutritional strategies for PCOS patients in relation to the desired outcomes. A preliminary meta-analysis published in 2023 by Khalid et al. [10] summarized data from 7 studies, indicating improvements in androgen profiles. However, since then, new trials have been published, and a more detailed exploration of the mechanisms—specifically the interplay between metabolic changes and hormonal shifts—is warranted.

## Aim

The primary objective of this meta-analysis is to assess whether the KD—in the form of VLCKD, LCKD, or modified KD —is associated with improvements in biochemical markers of hyperandrogenism in PCOS women with obesity or overweight. According to International Guidelines [1], most studies use total (TT) and free testosterone (FT) to assess biochemical hyperandrogenism in the diagnosis of PCOS; therefore, the primary outcomes of the present study were changes in TT and FT, independently of the method of measurement.

Secondarily, we explored whether KD protocols are associated with a change in luteinizing hormone/follicle stimulating hormone (LH/FSH) ratio. It is believed that PCOS women show a powerful LH secretion by the anterior pituitary due to a constitutional increase in the frequency and amplitude of gonadotropin releasing hormone (GnRH) pulses [11]. PCOS patients also exhibit decreased FSH levels, leading to an increased LH/FSH ratio, which in turn stimulates excess androgen production from ovarian theca cells and ultimately disrupts follicular development and ovulation [11]. For many years, an elevated LH/FSH ratio was used as a diagnostic marker for PCOS [12]. However, it is no longer recommended due to its limited clinical utility [13]. Nevertheless, it has been linked to TT levels [14] and proposed as a predictor of poor outcomes in Medically Assisted Reproduction [15]. Finally, the effectiveness of the KD in reducing body weight and visceral fat, as well as improving body composition and the glyco-lipid profile, was evaluated.

All available studies were considered, including randomized clinical trials (RCTs) and non-randomized prospective or retrospective observational studies. The outcomes analyzed include TT, Sex Hormones Binding Globulin (SHBG), FT, LH, FSH, the LH/FSH ratio, BMI, waist circumference (WC), fat mass, fasting glucose, fasting insulin, Homeostatic Model Assessment (HOMA)-insulin resistance (IR) index, total cholesterol, high-density lipoprotein (HDL) cholesterol, low-density lipoprotein (LDL) cholesterol and triglycerides levels.

An initial objective was to evaluate the effects of the KD on menstrual cycle regularity and reproductive outcomes; however, due to heterogeneity in study variables and methodology, a meta-analysis of these outcomes was not feasible.

## Methods

This research was registered on PROSPERO (CRD42023389506).

### Search strategy

A systematic search was conducted in five databases: MEDLINE (via PubMed), Embase, Cochrane Central Register of Controlled Trials (CENTRAL), Scopus, and Web of Science. No language restrictions were applied. The URL for the search strategy was: ("diet, ketogenic"[MeSH Terms] OR ("diet"[All Fields] AND "ketogenic"[All Fields]) OR "ketogenic diet"[All Fields] OR ("ketogenic"[All Fields] AND "diet"[All Fields])) AND "PCOS"[All Fields] AND ("VLCKD"[All Fields] AND ("ketogenic"[All Fields] AND ("polycystic ovary syndrome"[MeSH Terms] OR ("polycystic"[All Fields] AND "ovary"[All Fields] AND "syndrome"[All Fields]) OR "polycystic ovary syndrome"[All Fields])) AND ("VLCKD"[All Fields] AND ("polycystic ovary syndrome"[MeSH Terms] OR ("polycystic"[All Fields] AND "ovary"[All Fields] AND "syndrome"[All Fields]) OR "polycystic ovary syndrome" [All Fields])).

All studies published until March 1, 2025, were considered.

### Study population and interventions

Both RCTs and pre-post studies providing data for direct extraction or calculation of mean (SD) changes in the considered outcomes were included.

Inclusion criteria for participants were a diagnosis of PCOS based on the Rotterdam or European Society for Human Reproduction and Embryology/American Society for Reproductive Medicine (ESHRE/ASRM) criteria [16], aged 18 years or older, within reproductive age, and with a BMI of at least 25 kg/m^2^. Exclusion criteria were pregnancy and serious medical conditions requiring medical supervision.

Eligible studies involved participants treated with a KD, including VLCKD protocols (approximately 800 kcal/day) and LCKD protocols (1200–1400 kcal/day). Both protocols were characterized by low carbohydrate intake (≤ 50 g/day), 1–1.5 g of protein per kilogram of ideal body weight, and fat intake of less than 20–30 g/day. Modified KD regimens were also included.

Studies involving medical infertility treatments were included only if the data were collected during the dietary protocols and prior to the initiation of any medical treatment.

### Primary outcomes and effect measure

The primary outcome of this meta-analysis was the effect of the KD on TT, FT and SHBG levels compared to baseline. TT levels were included when measured using both the gold-standard method, tandem mass spectrometry (LC/MS), and immunologic assays. FT in the included studies was assessed by direct immunoassays. Additional hormonal outcomes included the KD’s impact on LH, FSH, and the LH/FSH ratio compared to baseline.

Although follow-up data were initially planned to include two observation periods (3 and 6 months), the studies analyzed had inconsistent or unique follow-up timelines; therefore, data closest to the 6-month follow-up were selected.

The effect measure was expressed as the mean difference (MD).

### Secondary outcomes

Secondary outcomes included BMI, WC, WHR, fat mass, fasting glucose, fasting insulin, HOMA-IR, total cholesterol, triglycerides, HDL cholesterol and LDL cholesterol. Reproductive outcomes considered were menstrual cycle regularization, ovulation, and pregnancy achievement. However, due to variability in evaluation methods, these outcomes were assessed qualitatively rather than quantitatively.

### Data extraction

Unpublished studies were identified through a search on ClinicalTrials.gov. Two researchers (EM and CA) independently conducted the identification of relevant abstracts, study selection, and data extraction from full-text articles. Extracted information included the author, year of publication, study design, diagnostic criteria, study population (sample size), number of participants in the intention-to-treat analysis, mean age and age range, inclusion and exclusion criteria, duration of dietary intervention (in days), type of KD, and outcome indicators (mean and standard deviation or SEM; success rate for categorical variables). If articles lacked sufficient information, the authors were contacted for additional data. Discrepancies between the two investigators were resolved discussion with a third author (GV).

### Synthesis strategy

Heterogeneity among studies was assessed using the I^2^ statistic, which quantifies the percentage of variation across studies. Since heterogeneity was significant, a random-effects model was applied to calculate the effect size, assuming that different studies estimated related but distinct interventions. All analyses were performed using StataCorp software, version 18 (Stata Statistical Software: Release 18. College Station, TX: StataCorp LLC).

### Quality assessment

The quality of included studies was evaluated using the following tools:

- Cochrane’s Risk of Bias Tool for Non-Randomized Studies (ROBINS-I) [17] for RCTs;
- NIH Quality Assessment Tool for Before-After (Pre-Post) Studies With No Control Group [18] for pre-post studies.

## Results

### Description of available studies

**Fig. 1:**
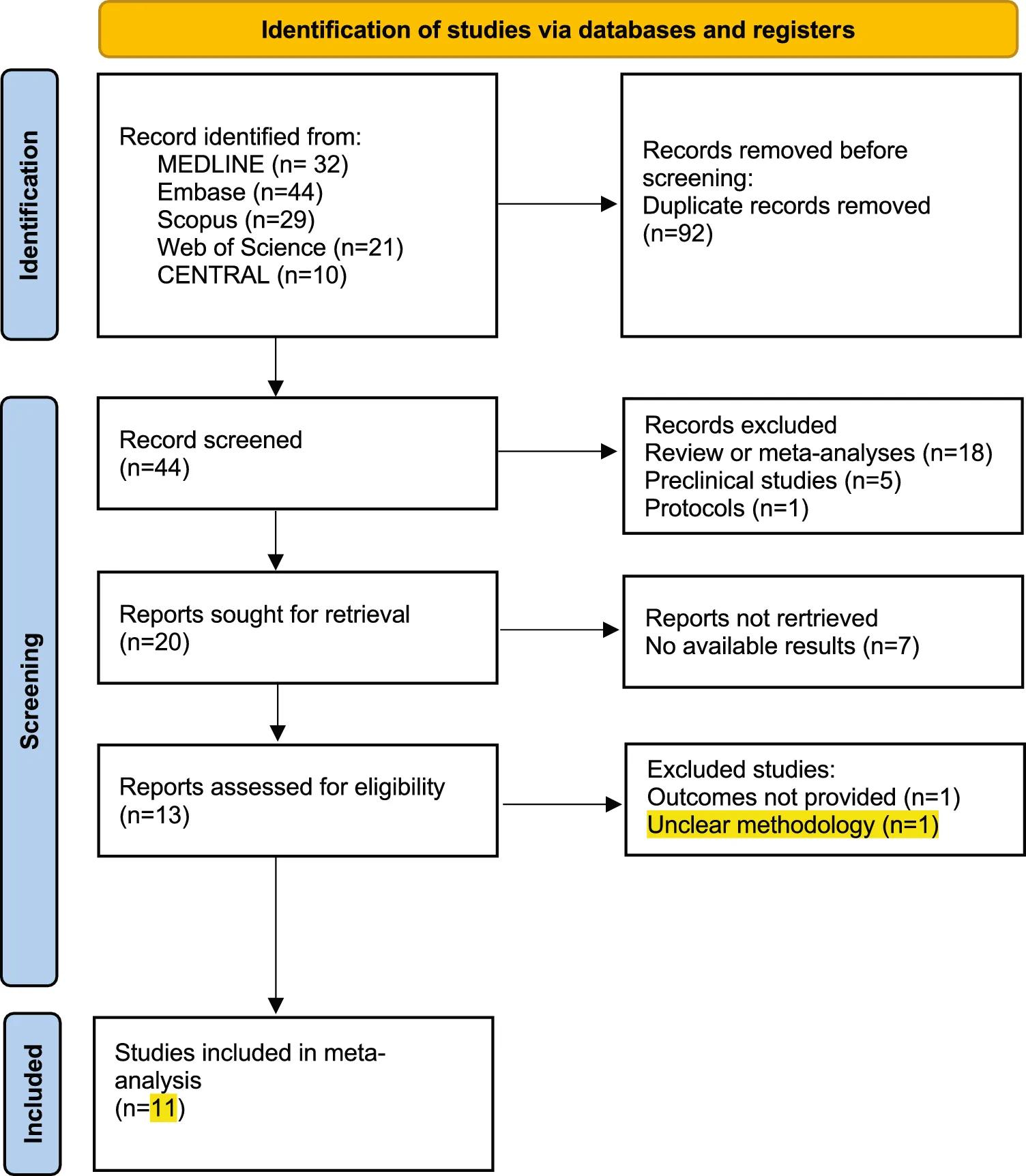
Study selection according to the PRISMA flowchart

The search identified 134 potentially relevant citations. Figure 1 illustrates the study selection process, conducted in accordance with the Preferred Reporting Items for Systematic Reviews and Meta-Analyses (PRISMA) guidelines [19].

The characteristics of the included studies are summarized in Table 1, while Table 2 provides a detailed overview of the dietary protocols used across different studies. The quality assessment of RCTs and pre-post studies is reported in Tables 3 and 4, respectively. The Mavropoulos et al. (2005) study [25] was removed from the quantitative analysis due to the small sample size (n = 5), the broad inclusion criteria (“and/or”), the lack of analytical detail regarding androgen assays and the occurrence of pregnancies during the study period.

**Table 1:** Characteristics of the included studies

Author, year	Study design	Sample size	Mean age (range)	BMI kg/m2	PCOS diagnosis	Diet	Androgens measurement	Main findings
Cincione et al., 2021 [20]	Pre-post, prospective	17	28.5 years, 18–45	> 25	Rotterdam Criteria	Modified KD	TT by immuno-chemiluminescent method, FT by radio-immunological assay	LH, TT and FT: statistically significant reduction FSH and SHBG: statistically significant increase
Cincione et al., 2023 [21]	RCT	73 KD vs. 71 Mediterranean diet	33.4 years, 18–45	> 25	ESHRE/ASRM Criteria	KD vs. low-calorie Mediterranean diet	TT by immuno-chemiluminescent method, FT by radio-immunological assay	The reduction of LH, LH/FSH ratio, FT, TT and the increase of FSH and SHBG were significantly greater in KD group than in Mediterranean Diet group
Li J et al., 2021 [22]	RCT	10 KD vs. 8 medications (polyene phos- phatidylcholine + COC)	31.1 years, 18–50	28–32	Rotterdam Criteria	Modified KD	NR	Significant reductions in menstrual cycle lenght, plasma estradiol, and progesterone levels in both groups (all p < 0.05); no significant between-group difference
Li M et al., 2025 [23]	Pre-post, retrospective with control group	35 KD vs. 35 low-calories diet + COC	31.2 years, 22–40	> 24	Rotterdam Criteria	flexible ketogenic	TT by electrochemiluminescence	In the KD group, significant reductions were observed in LH, LH/FSH ratio, and TT levels after treatment. No difference in TT change between KD and controls
Magagnini et al., 2022 [24]	Pre-post, retrospective	25	25.4 years, > 18	30–34.9	Rotterdam Criteria	VLCKD	NR	AMH serum levels significantly decreased, whereas progesterone and SHBG significantly increased
Mavropoulos et al., 2005 [25]	Pre-post, prospective	5	34.5 years, 18–45	> 27	based on history of chronic anovulation and/or hyperandrogenemia	LCKD	FT and TT by immunoassay and equilibrium ultrafiltration	Significant reductions in FT and LH, non-significant decrease in TT
Meneghini et al., 2023 [26]	Non-randomized controlled	42 VLCKD vs. 42 Mediterranean diet	33.9 years, 18–39	> 24.9	ESHRE Criteria	VLCKD vs. Mediterranean diet	TT by chemiluminescence immunoassay	Increased FSH/LH ratio and decreased TT levels, with higher differences in VLCKD vs. MD
Pandurevic et al., 2023 [27]	RCT	30: KD vs. 15 Mediterranean diet	26.4 years, 18–45	28–20	NIH Criteria	VLCKD vs. Mediterranean diet	TT by liquid chromatography-tandem mass spectrometry (LC–MS/MS)	No treatment significantly changed serum levels of TT, whereas SHBG significantly increased and FT significantly decreased in the KD group only. Changes in SHBG and FT were significantly higher in the KD vs. controls
Paoli et al., 2020 [28]	Pre-post, prospective	14	27.5 years, 18–45	> 25	Rotterdam Criteria	Ketogenic Mediterranean diet with phyoextracts (KEMEPHY)	TT by immuno-chemiluminescent method, FT by radioimmunological test	The LH/FSH ratio, LH, TT and FT, and DHEAS levels were significantly reduced. Estradiol, progesterone and SHBG increased
Rossetti et al., 2024 [29]	Pre-post, prospective	12	26.0 years	Mean 32 ± 6 also normal weight included	Rotterdam Criteria (Phenotype A)	Ketogenic diet	TT by chemiluminescent microparticle immunoassay	Menstrual cycles, FSH, LH, and progesterone improved significantly and independently of weight or fat loss
Sharifi et al., 2024 [30]	RCT	19 KD vs. 21 portfolio Moderate-carbohydrate diet	30.2 (18–45)	≥ 25 kg	Rotterdam Criteria	Ketogenic diet	fT by ELISA	Intragroup differences in the levels of LH, FSH, FT were statistically significant FSH and FT did not show a significant change between groups
Yang et al., 2022 [31]	Pre-post, prospective	55	29.7 years, 20–40	> 24	Rotterdam Criteria	Ketogenic diet	TT by chemiluminescence immunoassay	Increase in FSH, reduction in LH, LH/FSH and TT

AMH = Anti-Müllerian Hormone; FT = Free Testosterone; FSH = Follicle-Stimulating Hormone; BMI = Body Mass Index; KD = Ketogenic Diet; LH = Luteinizing Hormone; TT = Total Testosterone; SHBG = Sex Hormone Binding Globulin; PCOS = Polycystic Ovary Syndrome

**Table 2:** Characteristics of the dietary protocols used in the included studies

Author, year	Intervention (s)	Proteins in KD	Carbohydrates in KD	Fat in KD	Total caloric intake	Duration of KD
Cincione et al., 2021 [20]	Modified KD (isolated whey protein powder derived from milk, and animal protein sources such as meat, fish, or eggs)	1.1–1.2 g/kg/die ideal body weight	30 g/die	30 g/die	600 kcal/die	45 days
Cincione et al., 2023 [21]	Modified KD vs. low-calorie Mediterranean diet (MD)	KD: 1.1–1.2 g/kg/die ideal body weight MD: 20% of daily intake (especially fish and legumes)	KD: 30 g/die MD: 55% of daily intake (especially whole wheat)	KD: 30 g/die MD: 25% of daily intake (PUFA from olive oil, almonds and pistachios)	KD: 600 kcal/die MD: 500 kcal less than the individual daily caloric requirement	45 days
Li J et al., 2021 [22]	Modified KD: all kinds of oils, all kinds of meat (such as pork, beef, and fowl), eggs, seafood and vegetables	18–27% of energy	5%–10% of energy (≤ 50 g/day)	70–75% of energy	1300–1500 kcal/die	12 weeks
Li M et al., 2025 [23]	Flexible KD with a gradual increase of the fat-to-carbohydrate ratio vs. balanced dietary instructions including low-calorie diet	20%–27% of energy	3%–5% of energy (≤ 50 g/day)	70%–75% of energy	At or above the basal metabolic rate	12 weeks
Magagnini et al., 2022 [24]	VLCKD (high-biological protein derived from milk, peas, and whey)	As per VLCKD	As per VLCKD	As per VLCKD	Active stage: 600–800 kcal/die Re-education stage: 1200–1500 kcal/die Balanced diet stage: 1500–2000 kcal/die	12 weeks (each phase had a duration of 4 weeks)
Mavropoulos et al., 2005 [25]	LCKD; unlimited consumption of animal foods (meat, fish), prepared and fresh cheeses (up to 4 and 2 oz per day), unlimited eggs, and low carbohydrate vegetables (1 cupful per day)	NR	< 20 g/die	NR	NR	24 weeks
Meneghini et al., 2023 [26]	VLCKD vs. low-calorie MD	VLCKD: 50% of energy (67 g) MD: 15%	VLCKD: 20% of energy (25 g) MD: 55%	VLCKD: 30% of energy (50 g) MD: 30%	VLCKD: 800 kcal/die MD: 1400 kcal/die	120 days
Pandurevic et al., 2023 [27]	VLCKD Pronokal® method vs. MD	VLCKD and LCD: High-biological-value protein preparations obtained from cow’s milk, soy, eggs, green peas, and cereals MD: 15% of energy	VLCKD and LCD: < 50 g/die from vegetables MD: 55% of energy	VLCKD and LCD: 10 g of olive oil per day MD: 30% of energy	VLCKD: 600–800 kcal/die LCD: 1050–1400 kcal/die MD: 1200–1420 kcal/die	VLCKD for 8 weeks, followed by the LCD for another 8 weeks MD: 16 weeks
Paoli et al., 2020 [28]	Ketogenic Mediterranean diet with phytoextracts (KEMEPHY)	24.1% of energy	4.8% of energy	71.1% of energy	1600–1700 kcal/die	12 weeks
Rossetti et al., 2024 [29]	Ketogenic diet	1.3–1.4 g/kg	daily intake < 50 g	remaining caloric intake	isocaloric diet based on calculated total energy expenditure if normal weight; − 300/500 kcal of TEE if overweight	45 days
Sharifi et al., 2024 [30]	Ketogenic diet vs. Portfolio Moderate-carbohydrate diet (PMCD)	KD: 20% of energy from fish, poultry, meat, eggs PMCD: 20% (plant-based)	KD: 10% (< 30 g per day) PMCD: 40%	KD: 70% PMCD: 40%	500–700 kcal were removed from the total caloric intake	8 weeks
Yang et al., 2022 [31]	Ketogenic diet	18–27% of energy	5–10% of energy	70–75% of energy	Energy supply calculated by referring to the basal metabolism	12 weeks

KD = ketogenic diet; LCKD = low-calorie ketogenic diet; VLCKD = very low-calorie ketogenic diet; TEE = total energy expenditure

**Table 3:** Quality assessment of controlled studies according to the Cochrane’s risk of bias tool for randomized studies (ROBINS-I) [17]

	Random sequence generation	Allocation concealment	Blinding of participants and personnel	Blinding of outcome assessment	Incomplete outcome data	Selective reporting	Other bias
Cincione et al., 2023 [21]	-	?	-	?	-	?	-
Li J et al., 2021 [22]	+	?	-	?	+	+	-
Pandurevic et al., 2023 [27]	?	?	-	?	-	+	-
Sharifi et al., 2024 [30]	+	?	-	?	-	+	-

“- ” = low risk of bias, “ + ” = high risk of bias, “?” = unclear risk of bias

**Table 4:** Assessment of quality of pre-post studies according to NIH “Quality assessment tool for before-after (Pre-Post) studies with no control group” [18]

Study	NIH n.1	NIH n.2	NIH n.3	NIH n.4	NIH n.5	NIH n.6	NIH n.7	NIH n.8	NIH n.9	NIH n.10	NIH n.11	NIH n.12
Cincione et al., 2021 [20]	No	Yes	Yes	Yes	NR	Yes	Yes	No	Yes	Yes	No	NA
Li M et al., 2025 [23]	Yes	Yes	Yes	Yes	NR	Yes	Yes	No	Yes	Yes	No	NA
Magagnini et al., 2022 [24]	Yes	Yes	Yes	Yes	NR	Yes	Yes	No	Yes	Yes	No	NA
Mavropoulos et al., 2005 [25]	Yes	No	No	Yes	NR	Yes	No	No	No	Yes	No	NA
Meneghini et al., 2023 [26]	Yes	Yes	Yes	Yes	NR	Yes	Yes	Yes	NR	Yes	Yes	NA
Paoli et al., 2020 [28]	Yes	Yes	Yes	Yes	NR	Yes	Yes	No	Yes	Yes	No	NA
Rossetti et al., 2024 [29]	No	Yes	Yes	Yes	Yes	Yes	Yes	No	No	Yes	No	NA
Yang et al., 2022 [31]	Yes	Yes	Yes	Yes	Yes	Yes	Yes	Yes	Yes	Yes	No	NA

1) Was the study question or objective clearly stated?

2) Were eligibility/selection criteria for the study population pre-specified and clearly described?

3) Were the participants in the study representative of those who would be eligible for the test/service/intervention in the general or clinical population of interest?

4) Were all eligible participants that met the pre-specified entry criteria enrolled?

5) Was the sample size sufficiently large to provide confidence in the findings?

6) Was the test/service/intervention clearly described and delivered consistently across the study population?

7) Were the outcome measures pre-specified, clearly defined, valid, reliable, and assessed consistently across all study participants?

8) Were the people assessing the outcomes blinded to the participants’ exposures/interventions?

9) Was the loss to follow-up after baseline 20% or less? Were those lost to follow-up accounted for in the analysis?

10) Did the statistical methods examine changes in outcome measures from before to after the intervention? Were statistical tests done that provided p values for the pre-to-post changes?

11) Were outcome measures of interest taken multiple times before the intervention and multiple times after the intervention (i.e., did they use an interrupted time-series design)?

12) If the intervention was conducted at a group level (e.g., a whole hospital, a community, etc.) did the statistical analysis take into account the use of individual-level data to determine effects at the group level?

### Meta-analysis of all studies

#### Primary outcomes

For all meta-analyses, significant heterogeneity among studies was observed; therefore, a random-effects model was used for effect size calculation.

Sensitivity analyses were conducted according to the study design (RCTs, prospective and retrospective) for all outcomes, and are reported in each Figure. For RCTs, “before” and “after” data regarding the KD arm of study were considered.

**Fig. 2:**
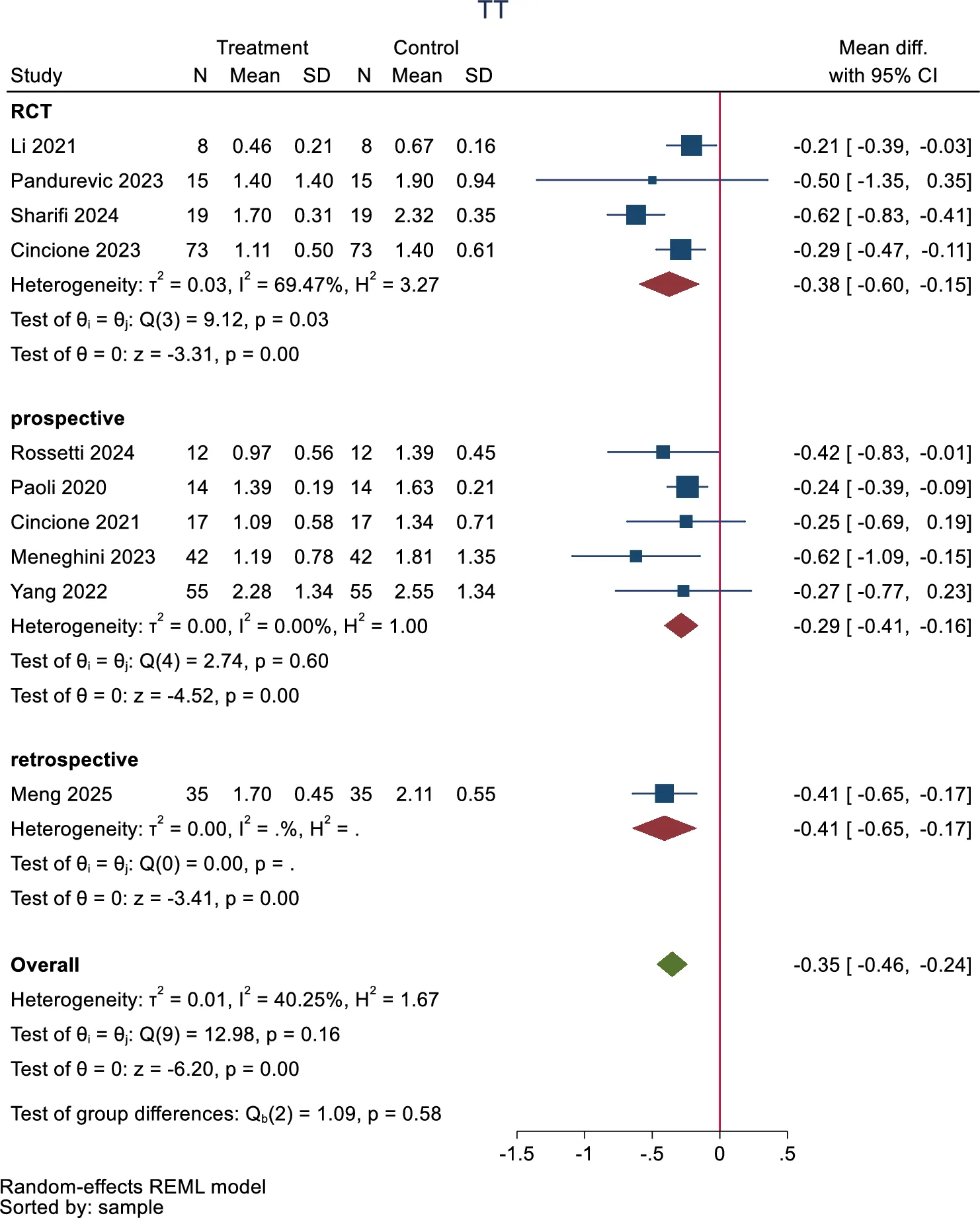
Mean differences with 95% Confidence Interval (CI) in total testosterone levels in patients with polycystic ovary syndrome undergoing a ketogenic diet protocol. The analysis includes both observational studies and controlled clinical trials (RCT)

**Fig. 3:**
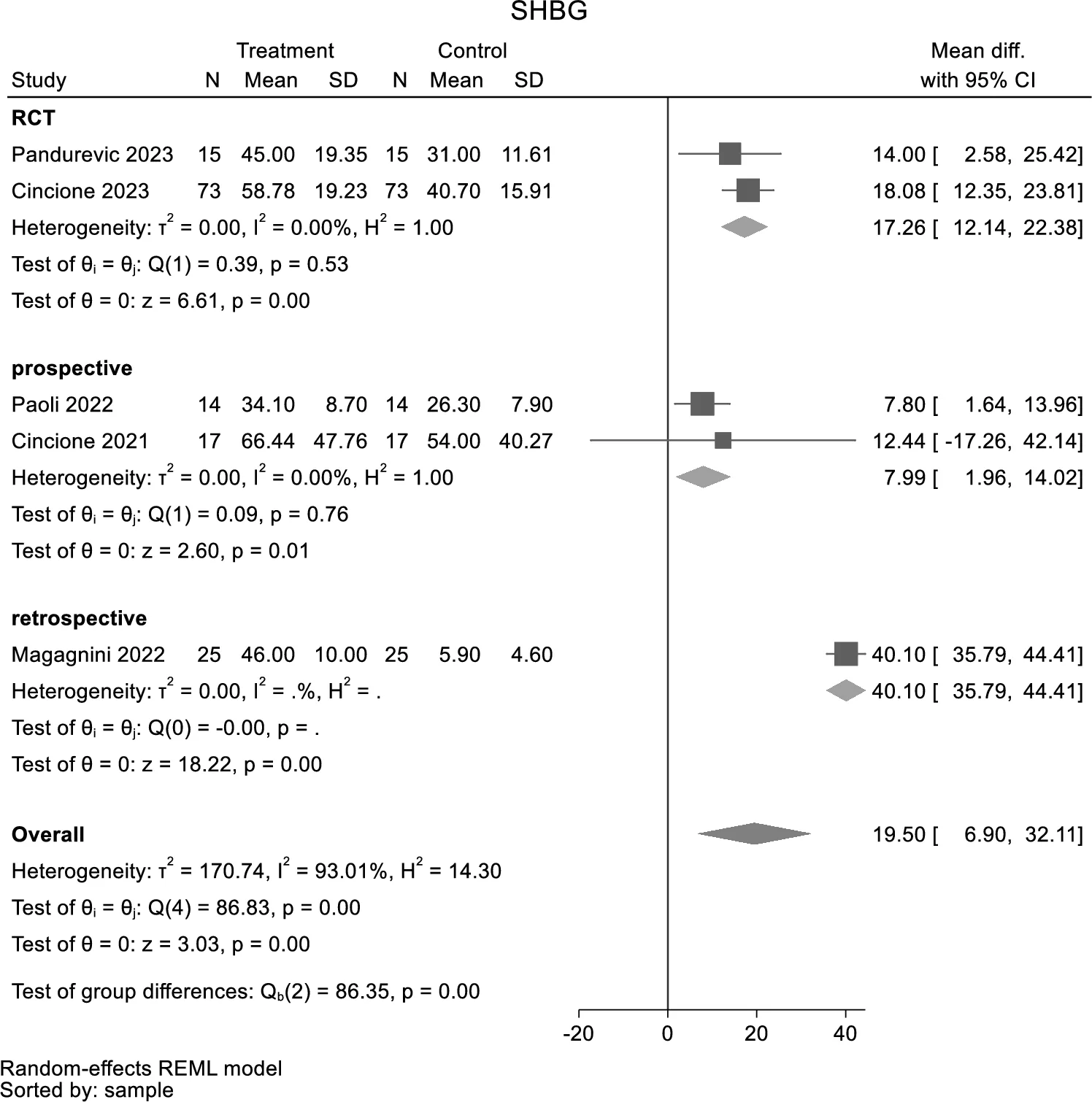
Mean differences with 95% Confidence Interval (CI) in SHBG (Sex Hormone Binding Globulin) levels in patients with polycystic ovary syndrome undergoing a ketogenic diet protocol. The analysis includes both observational studies and controlled clinical trials (RCT)

**Fig. 4:**
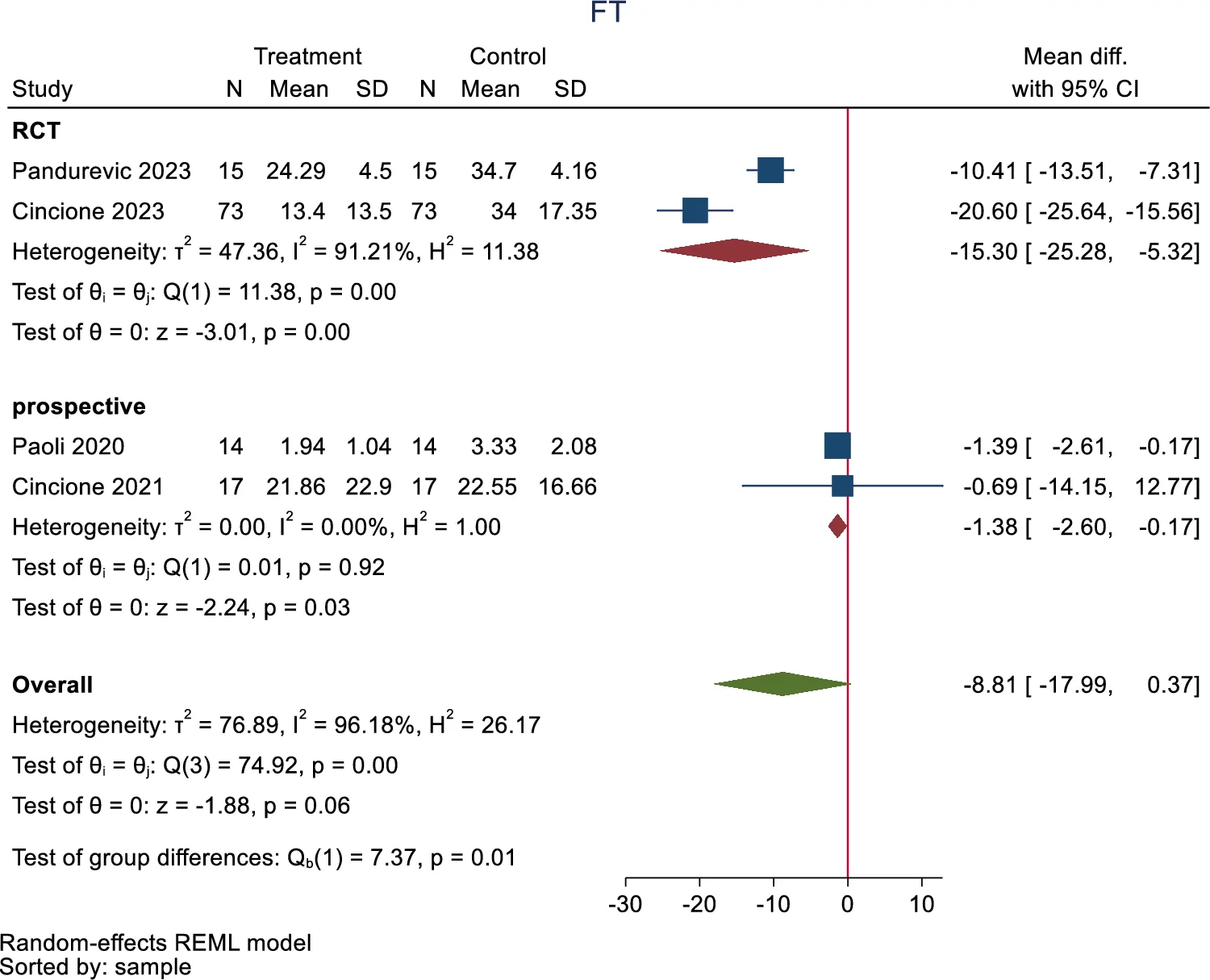
Mean differences with 95% Confidence Interval (CI) in free testosterone levels in patients with polycystic ovary syndrome undergoing a ketogenic diet protocol. The analysis includes both observational studies and controlled clinical trials (RCT)

The KD was found to be associated with a statistically significant reduction in TT (n = 290, MD -0.35, CI -0.46; -0.24) (Fig. 2) and a significant increase in SHBG levels (n = 144, MD 19.50, CI 6.90; 32.11) (Fig. 3), independently of study design. For each of the tested outcomes, a sensitivity analysis using the "leave-one-out" principle revealed that the overall effect estimate did not significantly change when each study was excluded (not shown). The reduction in FT was not statistically significant (n = 119, MD -8.81, CI -17.99; 0.37) (Fig. 4).

In order to investigate the factors possibly affecting the change in TT levels, we next performed meta-regression analyses. Although these meta-regressions can be limited by the small number of studies, neither the duration of the KD protocol, the change in BMI, the change in fasting insulin levels nor the change in LH/FSH ratio, were able to significantly affect the change in TT levels observed over time. Similarly, neither the change in fasting insulin levels nor the change in BMI affected the decrease in FT levels (not shown).

**Fig. 5:**
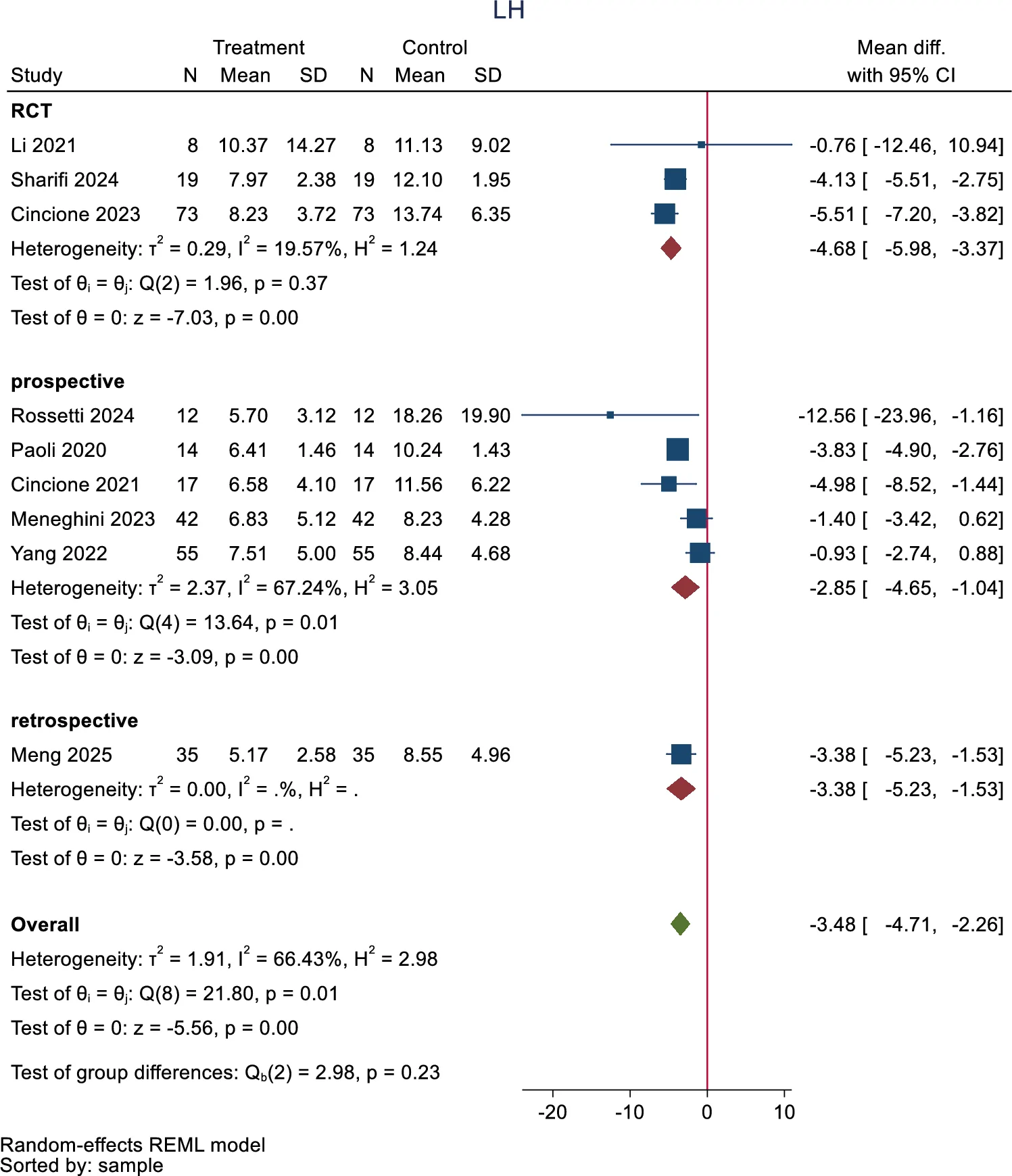
Mean differences with 95% Confidence Interval (CI) in LH (Luteinizing Hormone) levels in patients with polycystic ovary syndrome undergoing a ketogenic diet protocol. The analysis includes both observational studies and controlled clinical trials (RCT)

**Fig. 6:**
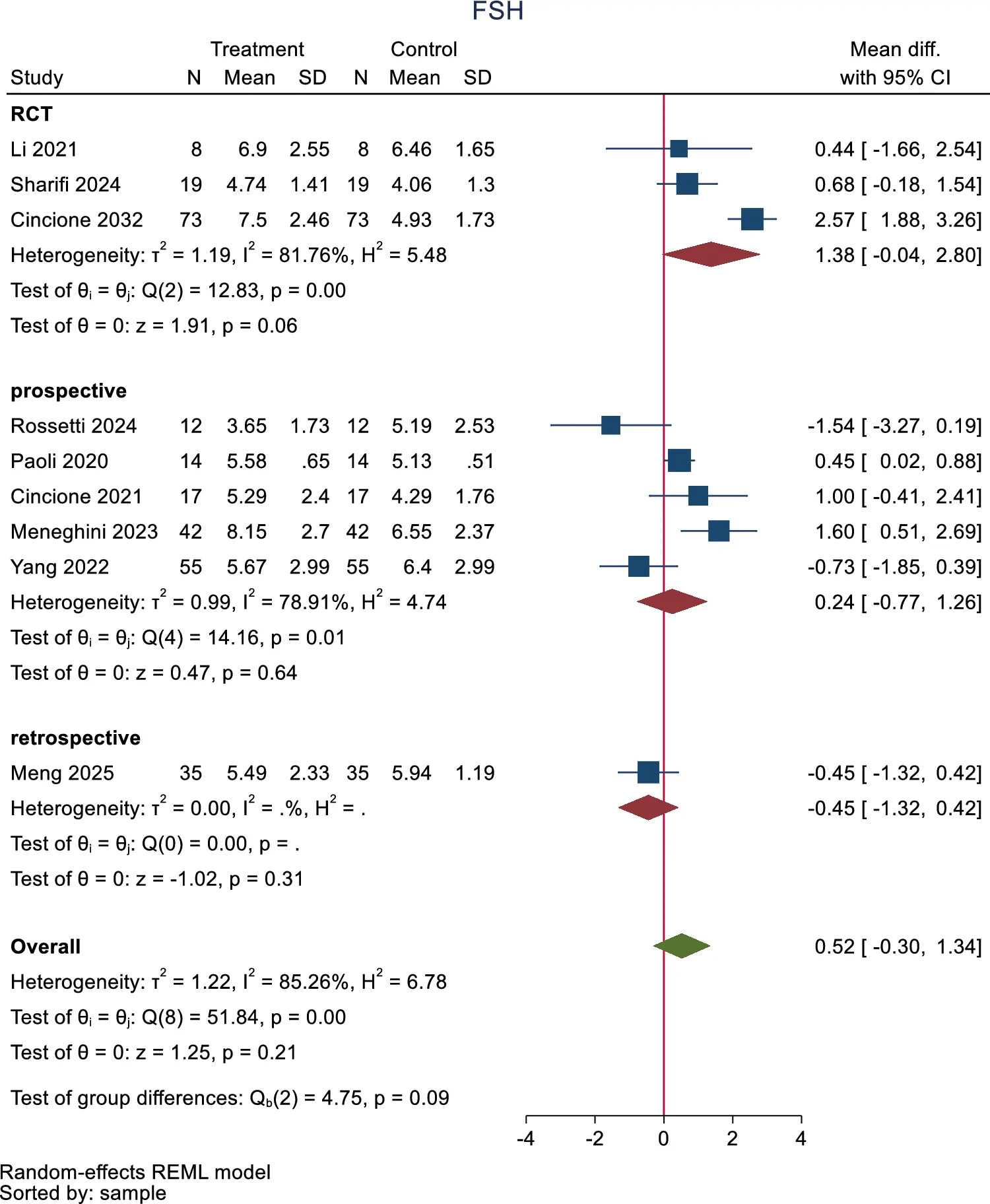
Mean differences with 95% Confidence Interval (CI) in FSH (Follicle-Stimulating Hormone) levels in patients with polycystic ovary syndrome undergoing a ketogenic diet protocol. The analysis includes both observational studies and controlled clinical trials (RCT)

**Fig. 7:**
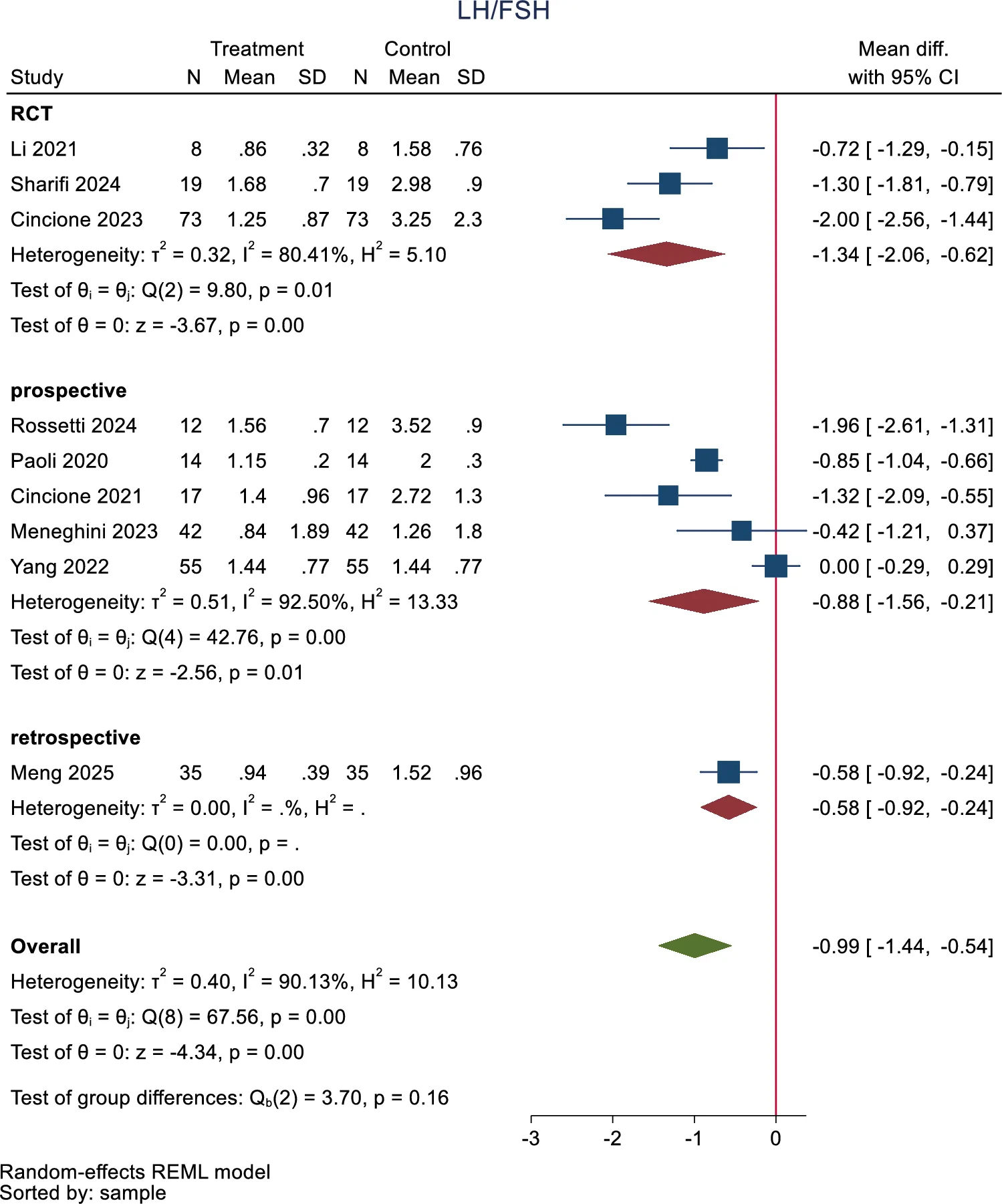
Mean differences with 95% Confidence Interval (CI) in LH/FSH ratio (Luteinizing Hormone/Follicle-Stimulating Hormone) levels in patients with polycystic ovary syndrome undergoing a ketogenic diet protocol. The analysis includes both observational studies and controlled clinical trials (RCT)

In addition, the KD associated with a significant reduction in LH levels (n = 275, MD -3.48, CI -4.71; -2.26) (Fig. 5) and, even though it did not reach a statistical significance, an increase in FSH levels was also observed (n = 275, MD 0.52, CI -0.30; 1.34) (Fig. 6). Therefore, the LH/FSH ratio (n = 275, MD -0.99, CI -1.44; -0.54) resulted significantly reduced overtime (Fig. 7). Sensitivity analysis using the "leave-one-out" principle did not significantly change the result for the reported outcomes (not shown). Noteworthy, meta-regression analysis revealed that reductions in insulin and BMI did not affect the change in the LH/FSH ratio (not shown).

### Secondary outcomes

Regarding clinical parameters, the KD associated with a significant reduction in BMI (n = 327, MD -4.24, CI -4.88; -3.61) (Supplementary Fig. 1), WC (n = 187, MD -8.42, CI -10.72; -6.11) (Supplementary Fig. 2), WHR (n = 171, MD -0.02, CI -0.03; -0.01) (Supplementary Fig. 3), and fat mass (n = 170, MD -7.74, CI -9.54; -5.95) (Supplementary Fig. 4), independently of study design. A significant reduction in fasting glucose (n = 260, MD -7.80, CI -11.39; -4.21) (Supplementary Fig. 5), fasting insulin (n = 197, MD -9.11, CI -14.79; -3.43) (Supplementary Fig. 6), HOMA-IR (n = 264, MD -2.49, CI -3.71;-1.27) (Supplementary Fig. 7), total cholesterol (n = 229, MD -15.95, CI -31.04; -0.86) (Supplementary Fig. 8), and triglycerides levels (n = 229, MD -44.29, CI -57.31; -31.27) (Supplementary Fig. 9) was also observed to be associated to KD. With KD we observed also a marginally significant increase HDL cholesterol (n = 229, MD 4.53, CI 0.24; 8.81) (Supplementary Fig. 10), and a non-significant decrease in LDL cholesterol (n = 229, MD -8.47, CI -24.30; 7.36) (Supplementary Fig. 11).

When meta-analyzing the data according to the study design, we confirmed all the observed associations in RCTs, with the exception of the increase in HDL level (Supplementary Fig. 10). We also found that, in prospective studies, the reduction in fasting insulin and total cholesterol were no more significant (Supplementary Fig. 6 and 8). The latter, along with HOMA-IR, resulted no more significantly reduced in retrospective studies (Supplementary Fig. 7 and 8). Among secondary outcomes, sensitivity analysis using the "leave-one-out" principle did not significantly changed the findings with the exception of the meta-analyses of total, HDL and LDL Cholesterol (not shown).

### Qualitative analysis of reproductive outcomes

Due to significant heterogeneity in the assessment criteria for reproductive outcomes, a quantitative analysis could not be performed. Table 5 provides a summary of these results, showing that the KD was associated with a positive effect on fertility outcomes investigated in all studies.

**Table 5:** Reproductive outcomes reported in the included studies. AMH = Anti-Müllerian Hormone

Author, year	Reproductive outcome	Methods	Results
Cincione et al., 2021 [20]	Menstrual regularity, pregnancy	Diary Pregnancy: NR	5/17 patients had a natural reappearance of a regular menstrual cycle after years of amenorrhea and 12/17 patients benefited from menstrual cycle regularity restoration; 5/12 achieved natural pregnancy after numerous previous unproductive attempts
Li J et al., 2021 [22]	Menstrual cycle duration	Diary	Significant reduction in menstrual cycle, but no significant difference vs. controls
Magagnini et al., 2022 [24]	AMH and progesterone levels	Blood testing on the 21st day of the menstrual cycle (Cobas Elecsys)	% of patients with progesterone levels at the 21st day of menstrual cycle > 15.9 ng/mL (sign of ovulation)
Mavropoulos et al., 2005 [25]	Pregnancy	NR	2/5 patients became pregnant despite previous infertility problems
Meneghini et al., 2023 [26]	Menstrual regularity	Diary	VLCKD success rate in 95% of cases, MD success rate in 60% of cases with statistically significant difference
Pandurevic et al., 2023 [27]	Ovulation	Pelvic ultrasound, progesterone and E2 test (ovulation occurred if a dominant follicle > 12 mm or corpus luteum seen on ultrasound + progesterone and E2 > 8 and 100 pg/mL respectively)	At baseline, 38.5% of participants in the experimental group and 14.3% of participants in the control group had ovulation, which increased to 84.6% (P = 0.031) and 35.7% (P > 0.05) at the end of the study, respectively
Yang et al., 2022 [31]	Menstrual regularity	Diary	66.7% of the hyperuricaemia and 53.6% of the non-hyperuricaemia group had resumed normal menstruation

VLCKD = Very Low-Calorie Ketogenic Diet; MD = Mediterranean Diet

In the study by Cincione and colleagues, out of 17 patients, 5 experienced a natural resumption of their menstrual cycle after years of amenorrhea, and 12 achieved spontaneous pregnancies following numerous unsuccessful attempts [20]. In the study by Li J and colleagues, the duration of the menstrual cycle was significantly reduced at follow-up compared to baseline in both groups, with no statistically significant difference observed between patients on the KD (32.5 ± 2.67 days vs. 72.50 ± 33.17 days) and controls (33.0 ± 33.17 days vs. 63.00 ± 19.75 days) [22].

In the study by Magagnini et al., serum progesterone levels measured on the 21st day increased after VLCKD, with 100% of patients achieving values indicative of ovulation (> 15.9 ng/ml, as reported by the authors) at follow-up [24]. The study by Mavropoulos and colleagues reported that 2 out of 5 women who completed the dietary protocol achieved pregnancy despite a previous diagnosis of infertility [25]. Meneghini and colleagues evaluated menstrual cycle regularity and found that it was achieved in 95% of patients undergoing the KD, compared with 60% of cases of those following a Mediterranean protocol [26]. Lastly, in the work of Pandurevic and colleagues, a significant increase in ovulation (+ 46.1%) was observed after VLCKD, compared to a 21.4% increase following a Mediterranean diet [27].

### Meta-analysis of RCTs: total testosterone

**Fig. 8:**
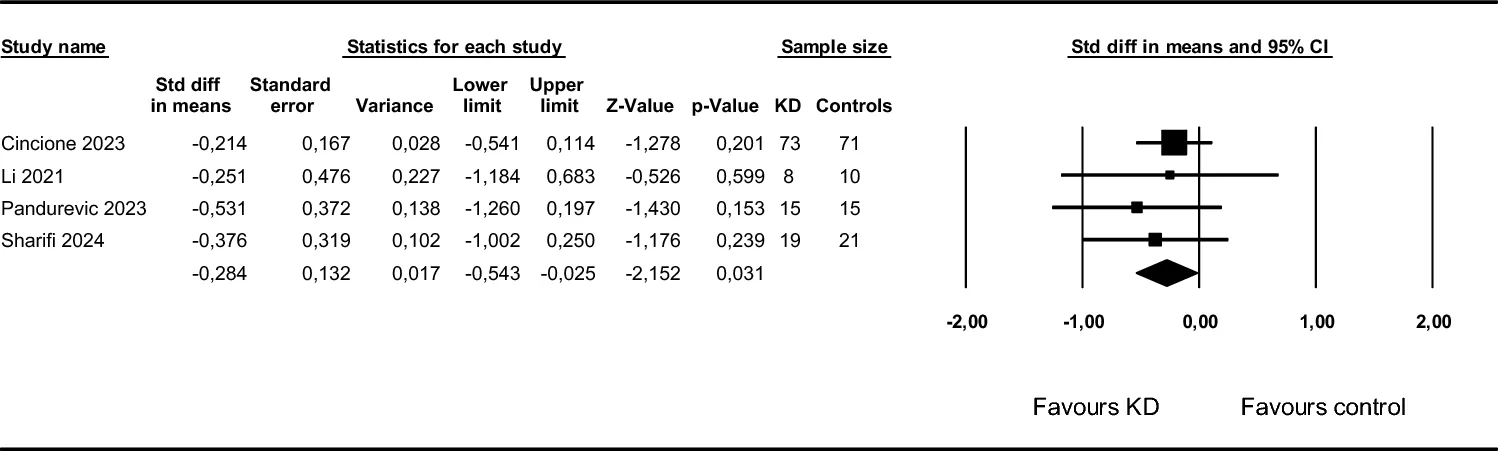
Standardized mean differences with 95% Confidence Interval (CI) in total testosterone levels in patients with polycystic ovary syndrome undergoing a ketogenic diet protocol or another type of intervention. Only controlled clinical trials (RCTs) are included

The only primary outcome reported in at least 3 RCTs including a control group was TT. In three of the 4 trials, the control group consisted of patients following an hypocaloric Mediterranean diet protocol [21, 27, 30]. In one study, the control group received a pharmacological intervention (Phosphatidylcholine combined with a hormonal contraceptive) [22]. The KD showed a moderate, statistically significant benefit on TT levels compared to the control intervention, with a high heterogeneity (n = 115 vs. 117, SMD -0.284, CI -0.543, -0.035; p = 0.031) (Fig. 8).

## Discussion

Our key findings suggest that, in PCOS women with obesity or overweight, a KD significantly reduces TT levels, whereas the reduction in FT was not statistically significant. Notably, our results differ partly from those of Khalid et al. [10]; by not including the RCT by Pandurevic et al. [27], they reported a significant reduction in FT levels but did not explore the change in TT. Moreover, by including a larger number of trials and utilizing meta-regression, our study demonstrates that the reduction in TT occurs independently of KD-mediated improvements in metabolic parameters (change in BMI and insulin levels) and/or in the hypothalamic-pituitary axis (reduction in the LH/FSH ratio). This novel finding suggests a direct role of nutritional ketosis on ovarian physiology and steroidogenesis, which might specifically mediate the observed decrease in T levels by KD. While these results are promising, they should be interpreted with caution given the heterogeneity and limited quality of the primary studies included.

PCOS is a highly prevalent endocrine condition affecting women of reproductive age. While its pathogenesis is complex and multifactorial, it’s widely acknowledged that overweight and obesity play a significant role, at least in certain phenotypes of the syndrome [32]. It has been previously demonstrated that a body weight reduction of approximately 10% can be sufficient to restore ovulation regularity, thereby improving fertility and other syndrome-related symptoms [3]. Therefore, the aim of the present work was to evaluate all available evidence supporting the role of the KD in PCOS, with a particular focus on ovarian function.

Among the meta-analyzable variables, TT emerged as significantly and independently reduced by KD in the meta-analysis of all included studies. This finding was further strengthened in the meta-analysis of TT, which included 4 RCTs with control arms. Here, the KD proved superior in reducing TT levels compared to other types of interventions (hypocaloric Mediterranean diet or the use of a COC). It’s important to note that the non-significant reduction in FT was also contributed to by an increase in SHBG. In PCOS, SHBG level measurement is also garnering renewed clinical interest due to the fact that a level below 33.4 nmol/l was identified to predict a pathological Liver Fat Score [33]. Therefore, an increase in SHBG by KD might also improve Metabolic dysfunction-associated steatotic liver disease (MASLD). Accordingly, weight loss and improved insulin sensitivity, both common outcomes of a KD, are known to improve hepatic dysfunction [34].

In recent years, several studies have been suggesting that KD protocols—differing in duration, composition and calories intake—have beneficial effects on hormonal parameters in women of reproductive age with PCOS and concomitant obesity/overweight. Indeed, changes in hormonal levels appear stronger than those obtained with other protocols, and independent of weight loss. In particular, as noted by Cincione et al., the benefits of KD extend beyond anthropometric measurements and metabolic indices (such as BMI, glucose, insulin, and HOMA-IR) to include FT, LH, FSH and the LH/FSH ratio [21]. In their study, the advantages of the KD were more pronounced compared to those following a Mediterranean diet, and these differences remained significant even after adjusting for variations in absolute BMI [21]. Similar results were obtained by Pandurevic et al., in which a VLCKD protocol conducted with the Pronokal® method was compared with Mediterranean LCD [27]. These findings possibly identify a specific role for nutritional ketosis, which may act on the pathogenetic mechanisms of PCOS, reducing insulin and IGF-1 levels and thus blocking their stimulating effect on ovarian and adrenal androgen production.

KD was also associated with a significant reduction in the LH/FSH ratio. As previously mentioned, In the pathogenesis of PCOS, there is also a change in the hypothalamic secretion pattern of GnRH, due to various mechanisms, including the alteration of negative feedback related to ovarian estrogens and insulin-induced stimulation [35]. The increase in GnRH pulses leads to an overproduction of LH compared to FSH, resulting in a reversal of their ratio. Although it does not fall among the diagnostic criteria, women with PCOS often exhibit a reversed LH/FSH ratio, two or three times higher than in healthy women, which is generally between 1 and 2. This alteration is responsible for the failure of follicular maturation, anovulation, and LH-induced hyperstimulation of the theca cells, resulting in hyperandrogenism [36, 37]. From the results of our meta-analysis, it emerged that, both considering all studies and only RCTs, the KD protocol is associated with a statistically significant reduction in LH, accompanied by a reduction in the LH/FSH ratio. Potential mechanisms responsible for KD-associated improvement of LH/FSH ration could be the improvement in insulin sensitivity or the reduced AMH level. In fact, previous researches showed a positive correlation between IR and LH levels or the LH/FSH ratio [38] with AMH serving as an mediating factor [39]. 

The study of the effect of the KD on reproductive outcomes has been more complex and has produced non-meta-analyzable results, due to the selection of heterogeneous and poorly comparable outcomes of the studies (Table 5). In addition, the presence of numerous confounding factors, including, foremost, the reproductive condition of the partner, were not taken into account in the considered studies. For all these reasons, we decided to present a qualitative synthesis of the results on reproductive outcomes. Overall, the KD was associated with a positive effect on fertility outcomes investigated in all studies. 

Despite the inherent limitations of a meta-analysis, we employed meta-regression to investigate the potential mediating factors behind the KD’s positive effect on hyperandrogenism. It was particularly interesting to observe that the effect on TT appeared to be independent not only of protocol duration and metabolic changes (specifically, reductions in BMI and insulin levels), but also of the reduction in the LH/FSH ratio. This finding aligns with emerging experimental evidence suggesting a direct effect of ketone bodies on ovarian function. For instance, a recent placebo-controlled RCT by Rittig et al. demonstrated that ketone supplementation, namely β-hydroxybutyrate, acutely lowers circulating androgen and glucose levels in women with PCOS, providing compelling support for a mechanism that operates beyond systemic metabolic improvements [40]. Whether the effect of ketosis is on androgen synthesis, metabolic, or clearance, this has yet to be elucidated.

Further bolstering this hypothesis, animal studies have also highlighted direct ovarian effects of ketosis. For example, research has shown that a KD alleviates the apoptosis of granulosa cells by inhibiting the activation of the cGAS-STING signaling pathway in PCOS [41]. In addition, β-hydroxybutyrate was demonstrated to suppress activation of the NLRP3 inflammasome in response to urate crystals and lipotoxic fatty acids in mice [42]. Similarly, studies exploring the role of short-chain fatty acids, such as butyric acid, have indicated an improvement in ovarian function and a decreased the expression of local inflammatory factors in the ovary of obese PCOS mice [43]. These experimental findings collectively suggest that ketone bodies, or the metabolic state of ketosis – especially in VLEKT -, may exert a direct influence on ovarian steroidogenesis and overall ovarian health, independently of the classic pathways involving weight loss or hypothalamic-pituitary regulation. Regarding human studies, in women with obesity undergoing a VLEKT protocol, multiple regression analysis identified the reduction in High-sensitivity C-reactive protein as the main predictor of MASLD improvement, explaining almost 66% of the variance [44]. Ultimately, this anti-inflammatory action may complement the reduction in hyperinsulinemia to restore ovarian function and improve the biochemical profile of women with PCOS.

Our meta-analysis has several limitations. Firstly, the limited number of analyzed studies, mostly of low quality, with small sample sizes, short durations, and only a minority represented by RCTs. Regarding the considered outcomes, there is often a lack of uniformity in the methods of biochemical parameter measurement. This is particularly important for determining T concentrations, where the gold standard method is LC/MS, which is limited by its availability and high costs. In several included studies, FT was obtained by direct radioimmunological or ELISA assays. These methods often exhibit limited accuracy and poor precision and sensitivity, thus reducing the strength of the results. Concerning the variables considered in various studies, the role of physical activity has not been evaluated in patients undergoing KD, even though it constitutes a first-line therapy in PCOS management. 

## Conclusions

In conclusion, while our meta-analysis indicates a reduced biochemical hyperandrogenism—particularly a reduction in TT—and a reduction in the LH/FSH ratio among women with PCOS following a KD, the evidence remains preliminary. Our findings also suggest a specific effect of ketosis on ovarian function acting alongside metabolic improvements, but a direct causal link cannot be definitively established. Owing to the low quality and heterogeneous nature of the available studies, including both RCTs and observational data, our conclusions must be viewed as hypothesis-generating. Larger, high-quality RCTs are essential to consolidate this evidence and determine clinical efficacy.

## Supplementary Information

Below is the link to the electronic supplementary material.

Supplementary file1

Supplementary file2

Supplementary file3

Supplementary file4

Supplementary file5

Supplementary file6

Supplementary file7

Supplementary file8

Supplementary file9

Supplementary file10

Supplementary file11

Supplementary file12

## Data Availability

The datasets generated during and/or analysed during the current study are available from the corresponding author on reasonable request.
