# Supplementary material for: Ketogenic diet as a therapeutic intervention for biochemical hyperandrogenism in PCOS women with obesity or overweight: a meta-analytic study
Source: J Endocrinol Invest. 2026 Jun 13;49(11):3185–202. doi: 10.1007/s40618-026-02924-1 (PMC13722772; doi:10.1007/s40618-026-02924-1)
Supplement: Supplementary file 1 [file 40618_2026_2924_MOESM10_ESM.pdf]

HOMA-IR

Study	Treatment N	Treatment Mean	Treatment SD	Control N	Control Mean	Control SD	Mean diff. with 95% CI
**RCT**							
Sharifi 2024	19	3.15	0.75	19	6.56	0.92	-3.41 [-3.94, -2.88]
Pandurevic 2023	27	2.11	1.26	27	2.74	1.48	-0.63 [-1.36, 0.10]
Cincione 2023	73	3.33	1.56	73	9.03	5.08	-5.70 [-6.92, -4.48]
Heterogeneity: τ^2 = 6.14, I^2 = 97.54%, H^2 = 40.58							-3.22 [-6.06, -0.37]
Test of $\theta_i = \theta_j$: Q(2) = 60.41, p = 0.00							
Test of θ = 0: z = -2.21, p = 0.03							
**prospective**							
Rossetti 2024	12	3.51	3.96	12	5.81	3.65	-2.30 [-5.35, 0.75]
Paoli 2020	14	2.32	0.13	14	2.85	0.15	-0.53 [-0.63, -0.43]
Cincione 2021	17	2.60	1.80	17	6.05	5.64	-3.45 [-6.26, -0.64]
Meneghini 2023	42	0.80	2.41	42	2.27	3.29	-1.47 [-2.70, -0.24]
Heterogeneity: τ^2 = 0.73, I^2 = 62.44%, H^2 = 2.66							-1.37 [-2.51, -0.22]
Test of $\theta_i = \theta_j$: Q(3) = 7.61, p = 0.05							
Test of θ = 0: z = -2.33, p = 0.02							
**retrospective**							
Magagnini 2022	25	1.50	0.80	25	5.60	0.80	-4.10 [-4.54, -3.66]
Meng 2025	35	2.75	0.94	35	4.04	1.78	-1.29 [-1.96, -0.62]
Heterogeneity: τ^2 = 3.86, I^2 = 97.89%, H^2 = 47.29							-2.71 [-5.46, 0.05]
Test of $\theta_i = \theta_j$: Q(1) = 47.29, p = 0.00							
Test of θ = 0: z = -1.93, p = 0.05							
**Overall**							-2.49 [-3.71, -1.27]
Heterogeneity: τ^2 = 3.03, I^2 = 97.44%, H^2 = 39.06							
Test of $\theta_i = \theta_j$: Q(8) = 398.99, p = 0.00							
Test of θ = 0: z = -4.00, p = 0.00							
Test of group differences: $Q_b(2)$ = 1.91, p = 0.38							

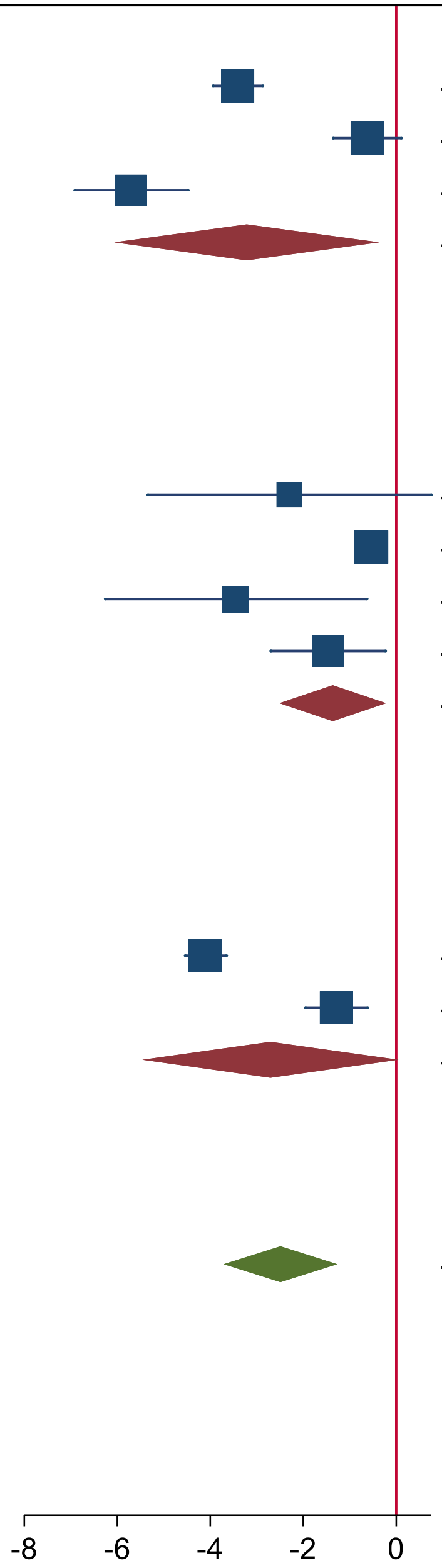

Random-effects REML model
Sorted by: sample
