# Supplementary material for: Ketogenic diet as a therapeutic intervention for biochemical hyperandrogenism in PCOS women with obesity or overweight: a meta-analytic study
Source: J Endocrinol Invest. 2026 Jun 13;49(11):3185–202. doi: 10.1007/s40618-026-02924-1 (PMC13722772; doi:10.1007/s40618-026-02924-1)
Supplement: Supplementary file 2 [file 40618_2026_2924_MOESM11_ESM.pdf]

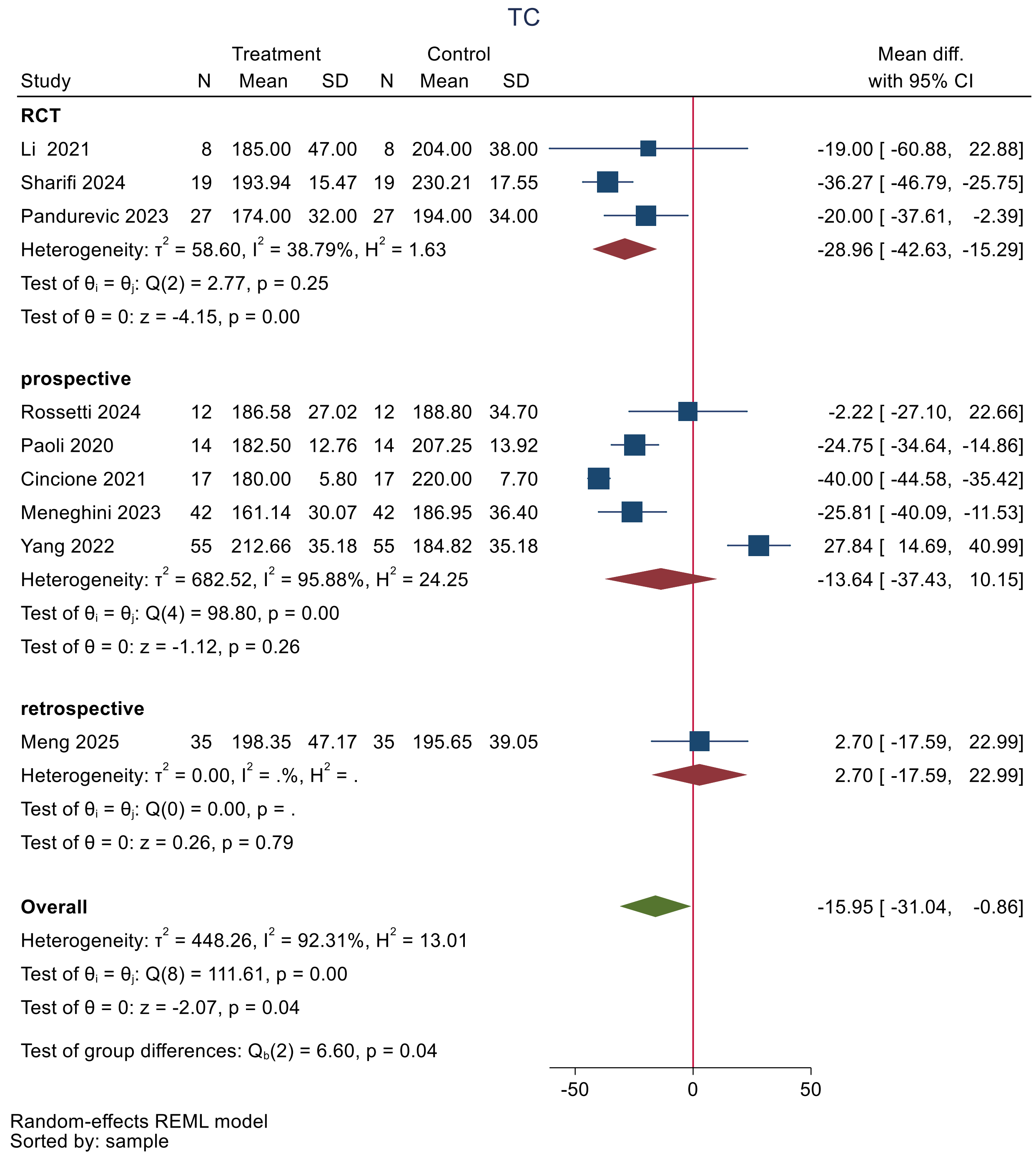
TC
Treatment
Control
Mean diff.
Study N Mean SD N Mean SD
with 95% CI
RCT
Li 2021 8 185.00 47.00 8 204.00 38.00 -19.00 [-60.88, 22.88]
Sharifi 2024 19 193.94 15.47 19 230.21 17.55 -36.27 [-46.79, -25.75]
Pandurevic 2023 27 174.00 32.00 27 194.00 34.00 -20.00 [-37.61, -2.39]
Heterogeneity: τ^2 = 58.60, I^2 = 38.79%, H^2 = 1.63 -28.96 [-42.63, -15.29]
Test of $\theta_i = \theta_j$: Q(2) = 2.77, p = 0.25
Test of θ = 0: z = -4.15, p = 0.00
prospective
Rossetti 2024 12 186.58 27.02 12 188.80 34.70 -2.22 [-27.10, 22.66]
Paoli 2020 14 182.50 12.76 14 207.25 13.92 -24.75 [-34.64, -14.86]
Cincione 2021 17 180.00 5.80 17 220.00 7.70 -40.00 [-44.58, -35.42]
Meneghini 2023 42 161.14 30.07 42 186.95 36.40 -25.81 [-40.09, -11.53]
Yang 2022 55 212.66 35.18 55 184.82 35.18 27.84 [14.69, 40.99]
Heterogeneity: τ^2 = 682.52, I^2 = 95.88%, H^2 = 24.25 -13.64 [-37.43, 10.15]
Test of $\theta_i = \theta_j$: Q(4) = 98.80, p = 0.00
Test of θ = 0: z = -1.12, p = 0.26
retrospective
Meng 2025 35 198.35 47.17 35 195.65 39.05 2.70 [-17.59, 22.99]
Heterogeneity: τ^2 = 0.00, I^2 = .%, H^2 = . 2.70 [-17.59, 22.99]
Test of $\theta_i = \theta_j$: Q(0) = 0.00, p = .
Test of θ = 0: z = 0.26, p = 0.79
Overall -15.95 [-31.04, -0.86]
Heterogeneity: τ^2 = 448.26, I^2 = 92.31%, H^2 = 13.01
Test of $\theta_i = \theta_j$: Q(8) = 111.61, p = 0.00
Test of θ = 0: z = -2.07, p = 0.04
Test of group differences: $Q_b(2)$ = 6.60, p = 0.04
-50
0
50
Random-effects REML model
Sorted by: sample
