# Supplementary material for: Ketogenic diet as a therapeutic intervention for biochemical hyperandrogenism in PCOS women with obesity or overweight: a meta-analytic study
Source: J Endocrinol Invest. 2026 Jun 13;49(11):3185–202. doi: 10.1007/s40618-026-02924-1 (PMC13722772; doi:10.1007/s40618-026-02924-1)
Supplement: Supplementary file 3 [file 40618_2026_2924_MOESM12_ESM.pdf]

TRIGLY

Study	Treatment N	Treatment Mean	Treatment SD	Control N	Control Mean	Control SD	Mean diff. with 95% CI
**RCT**							
Li 2021	8	147.00	71.00	8	216.00	147.00	-69.00 [-182.12, 44.12]
Sharifi 2024	19	214.42	31.97	19	272.04	34.72	-57.62 [-78.84, -36.40]
Pandurevic 2023	27	78.00	23.00	27	126.00	79.00	-48.00 [-79.04, -16.96]
Heterogeneity: τ^2 = 0.00, I^2 = 0.00%, H^2 = 1.00							-54.89 [-72.21, -37.58]
Test of $\theta_i = \theta_j$: Q(2) = 0.31, p = 0.86							
Test of θ = 0: z = -6.21, p = 0.00							
**prospective**							
Rossetti 2024	12	81.91	37.18	12	118.20	61.12	-36.29 [-76.77, 4.19]
Cincione 2020	14	165.63	23.91	14	204.60	35.43	-38.97 [-61.36, -16.58]
Cincione 2021	17	200.00	31.70	17	270.00	44.29	-70.00 [-95.89, -44.11]
Meneghini 2023	42	104.38	24.23	42	121.43	50.13	-17.05 [-33.89, -0.21]
Yang 2022	55	106.28	76.61	55	154.99	76.61	-48.71 [-77.34, -20.08]
Heterogeneity: τ^2 = 294.47, I^2 = 64.85%, H^2 = 2.85							-41.06 [-60.14, -21.99]
Test of $\theta_i = \theta_j$: Q(4) = 12.30, p = 0.02							
Test of θ = 0: z = -4.22, p = 0.00							
**retrospective**							
Meng 2025	35	123.99	56.68	35	165.63	69.97	-41.64 [-71.47, -11.81]
Heterogeneity: τ^2 = 0.00, I^2 = .%, H^2 = .							-41.64 [-71.47, -11.81]
Test of $\theta_i = \theta_j$: Q(0) = -0.00, p = .							
Test of θ = 0: z = -2.74, p = 0.01							
**Overall**							-44.29 [-57.31, -31.27]
Heterogeneity: τ^2 = 178.64, I^2 = 49.03%, H^2 = 1.96							
Test of $\theta_i = \theta_j$: Q(8) = 15.72, p = 0.05							
Test of θ = 0: z = -6.67, p = 0.00							
Test of group differences: $Q_b(2)$ = 1.29, p = 0.53							

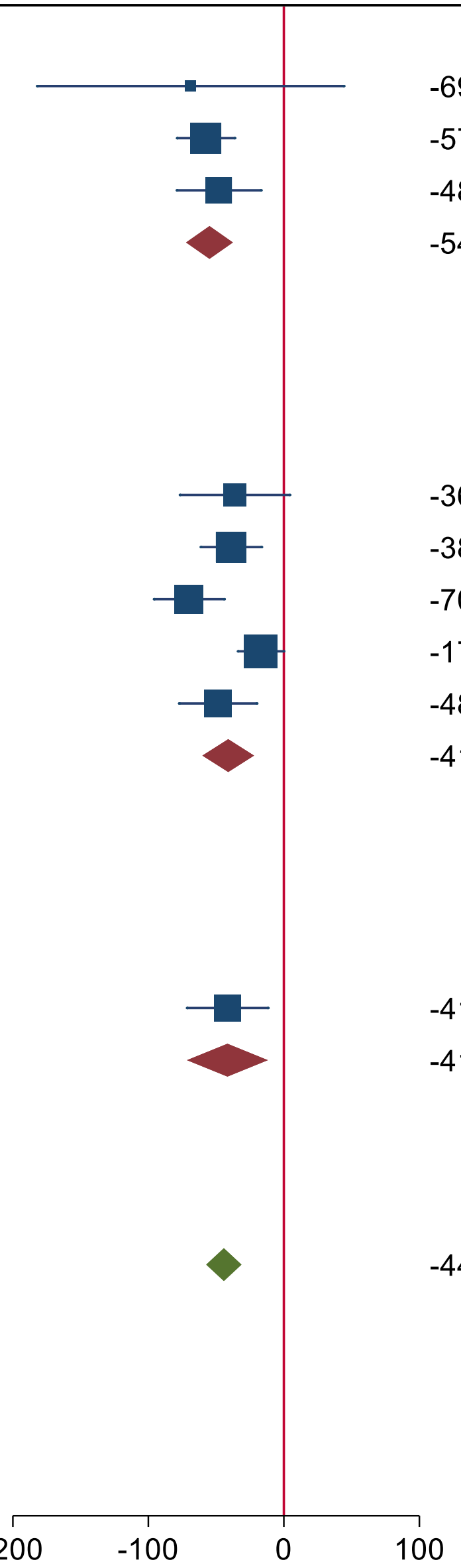

Random-effects REML model
Sorted by: sample
