# Supplementary material for: Ketogenic diet as a therapeutic intervention for biochemical hyperandrogenism in PCOS women with obesity or overweight: a meta-analytic study
Source: J Endocrinol Invest. 2026 Jun 13;49(11):3185–202. doi: 10.1007/s40618-026-02924-1 (PMC13722772; doi:10.1007/s40618-026-02924-1)
Supplement: Supplementary file 4 [file 40618_2026_2924_MOESM2_ESM.pdf]

BMI

Study	Treatment N	Treatment Mean	Treatment SD	Control N	Control Mean	Control SD	Mean diff. with 95% CI
**RCT**							
Li 2021	8	28.33	2.62	8	32.81	3.75	-4.48 [-7.65, -1.31]
Sharifi 2024	19	26.48	3.56	19	29.21	3.39	-2.73 [-4.94, -0.52]
Pandurevic 2023	27	29.30	3.90	27	33.90	3.80	-4.60 [-6.65, -2.55]
Cincione 2023	73	30.53	5.06	73	34.69	5.26	-4.16 [-5.83, -2.49]
Heterogeneity: $\tau^2 = 0.00$, $I^2 = 0.00\%$, $H^2 = 1.00$							-3.99 [-5.04, -2.93]
Test of $\theta_i = \theta_j$: Q(3) = 1.72, p = 0.63							
Test of $\theta = 0$: z = -7.40, p = 0.00							
**prospective**							
Rossetti 2024	12	28.71	6.46	12	32.18	5.97	-3.47 [-8.45, 1.51]
Paoli 2020	14	25.49	1.69	14	28.84	2.10	-3.35 [-4.76, -1.94]
Cincione 2021	17	29.93	5.47	17	31.84	5.85	-1.91 [-5.72, 1.90]
Meneghini 2023	42	25.91	2.74	42	31.23	4.25	-5.32 [-6.85, -3.79]
Yang 2022	55	26.00	5.00	55	31.50	5.25	-5.50 [-7.42, -3.58]
Heterogeneity: $\tau^2 = 0.81$, $I^2 = 41.56\%$, $H^2 = 1.71$							-4.32 [-5.59, -3.05]
Test of $\theta_i = \theta_j$: Q(4) = 6.55, p = 0.16							
Test of $\theta = 0$: z = -6.67, p = 0.00							
**retrospective**							
Magagnini 2022	25	29.00	1.00	25	32.80	1.00	-3.80 [-4.35, -3.25]
Meng 2025	35	23.82	3.27	35	29.72	4.33	-5.90 [-7.70, -4.10]
Heterogeneity: $\tau^2 = 1.74$, $I^2 = 79.11\%$, $H^2 = 4.79$							-4.67 [-6.70, -2.64]
Test of $\theta_i = \theta_j$: Q(1) = 4.79, p = 0.03							
Test of $\theta = 0$: z = -4.51, p = 0.00							
**Overall**							-4.24 [-4.88, -3.61]
Heterogeneity: $\tau^2 = 0.31$, $I^2 = 30.95\%$, $H^2 = 1.45$							
Test of $\theta_i = \theta_j$: Q(10) = 13.63, p = 0.19							
Test of $\theta = 0$: z = -13.14, p = 0.00							
Test of group differences: $Q_b(2) = 0.40$, p = 0.82							

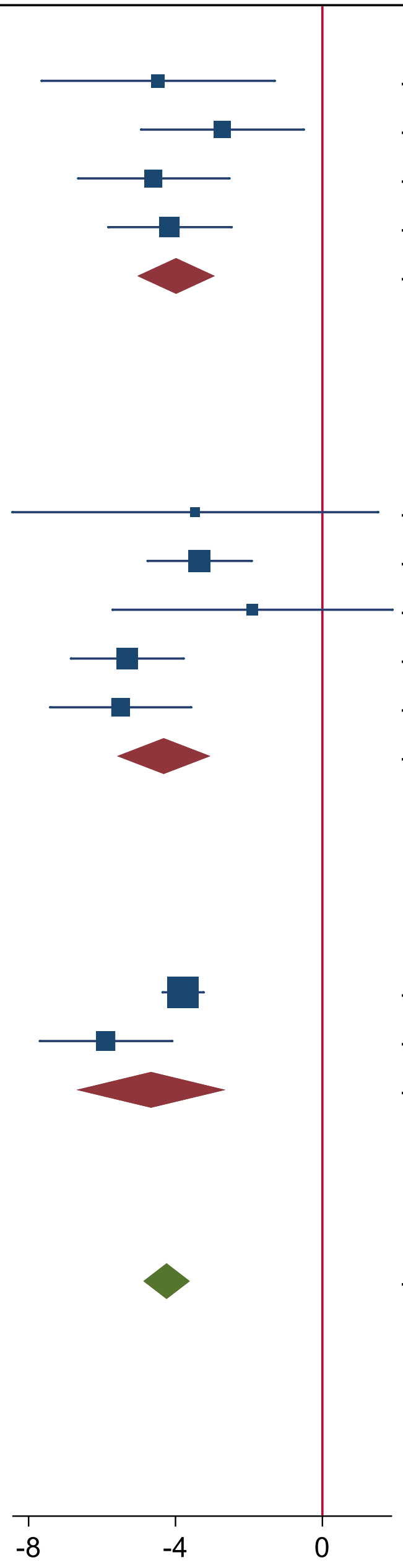

Random-effects REML model
Sorted by: sample
