# Supplementary material for: Ketogenic diet as a therapeutic intervention for biochemical hyperandrogenism in PCOS women with obesity or overweight: a meta-analytic study
Source: J Endocrinol Invest. 2026 Jun 13;49(11):3185–202. doi: 10.1007/s40618-026-02924-1 (PMC13722772; doi:10.1007/s40618-026-02924-1)
Supplement: Supplementary file 6 [file 40618_2026_2924_MOESM4_ESM.pdf]

LDL

Study	Treatment N	Treatment Mean	Treatment SD	Control N	Control Mean	Control SD	Mean diff. with 95% CI
**RCT**							
Li 2021	8	113.00	38.00	8	132.00	29.00	-19.00 [-52.12, 14.12]
Sharifi 2024	19	111.52	11.22	19	134.86	14.75	-23.34 [-31.67, -15.01]
Pandurevic 2023	27	109.00	29.00	27	117.00	25.00	-8.00 [-22.44, 6.44]
Heterogeneity: τ^2 = 50.28, I^2 = 45.39%, H^2 = 1.83							-17.47 [-29.36, -5.57]
Test of $\theta_i = \theta_j$: Q(2) = 3.25, p = 0.20							
Test of θ = 0: z = -2.88, p = 0.00							
**prospective**							
Rossetti 2024	12	107.09	23.70	12	113.90	34.15	-6.81 [-30.33, 16.71]
Cincione 2021	17	95.00	5.30	17	130.00	21.10	-35.00 [-45.34, -24.66]
Yang 2022	55	139.19	30.55	55	115.61	30.66	23.58 [12.14, 35.02]
Heterogeneity: τ^2 = 861.92, I^2 = 94.74%, H^2 = 19.00							-6.12 [-40.58, 28.35]
Test of $\theta_i = \theta_j$: Q(2) = 55.46, p = 0.00							
Test of θ = 0: z = -0.35, p = 0.73							
**retrospective**							
Meng 2025	35	122.96	31.32	35	115.61	29.39	7.35 [-6.88, 21.58]
Heterogeneity: τ^2 = 0.00, I^2 = .%, H^2 = .							7.35 [-6.88, 21.58]
Test of $\theta_i = \theta_j$: Q(0) = -0.00, p = .							
Test of θ = 0: z = 1.01, p = 0.31							
**Overall**							-8.47 [-24.30, 7.36]
Heterogeneity: τ^2 = 381.93, I^2 = 89.43%, H^2 = 9.46							
Test of $\theta_i = \theta_j$: Q(6) = 71.03, p = 0.00							
Test of θ = 0: z = -1.05, p = 0.29							
Test of group differences: $Q_b(2)$ = 6.88, p = 0.03							

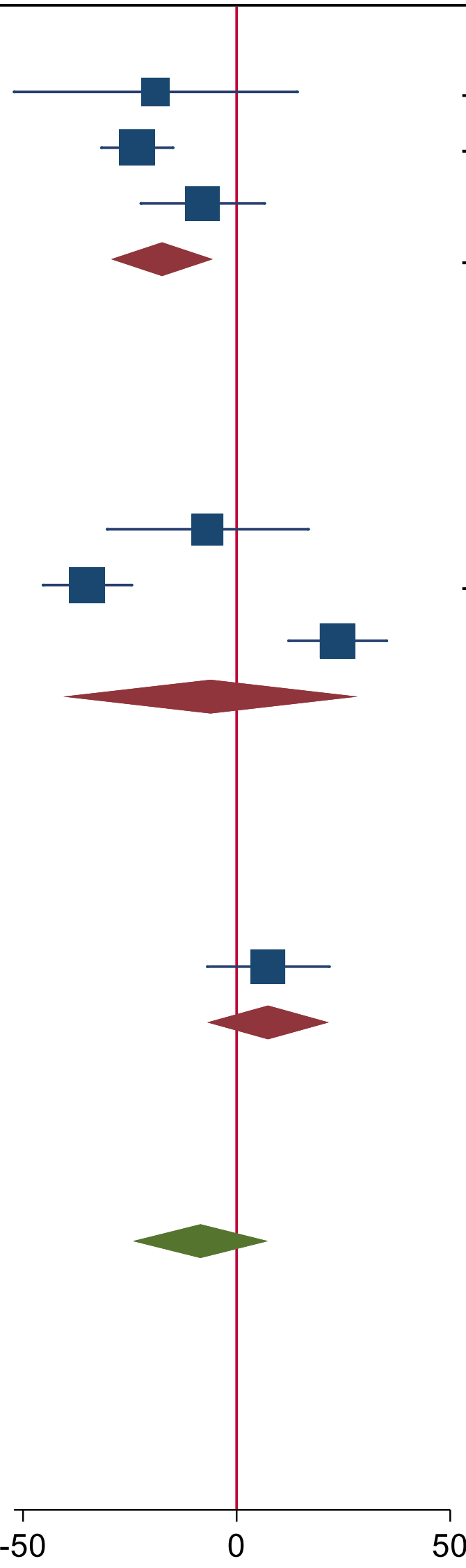

Random-effects REML model
Sorted by: sample
