# Supplementary material for: Ketogenic diet as a therapeutic intervention for biochemical hyperandrogenism in PCOS women with obesity or overweight: a meta-analytic study
Source: J Endocrinol Invest. 2026 Jun 13;49(11):3185–202. doi: 10.1007/s40618-026-02924-1 (PMC13722772; doi:10.1007/s40618-026-02924-1)
Supplement: Supplementary file 7 [file 40618_2026_2924_MOESM5_ESM.pdf]

## WC

| Study | Treatment N | Treatment Mean | Treatment SD | Control N | Control Mean | Control SD | Mean diff. with 95% CI |
|---|---|---|---|---|---|---|---|
| **RCT** | | | | | | | |
| Sharifi 2024 | 19 | 100.18 | 12.34 | 19 | 105.62 | 12.03 | -5.44 [ -13.19, 2.31] |
| Pandurevic 2023 | 27 | 87 | 9.2 | 27 | 98.2 | 5.6 | -11.20 [ -15.26, -7.14] |
| Cincione 2023 | 73 | 89.77 | 10.7 | 73 | 99.79 | 10.96 | -10.02 [ -13.53, -6.51] |
| Heterogeneity: $\tau^2$ = 0.00, $I^2$ = 0.00%, $H^2$ = 1.00 | | | | | | | -9.99 [ -12.50, -7.48] |
| Test of $\theta_i = \theta_j$: Q(2) = 1.67, p = 0.43 | | | | | | | |
| Test of θ = 0: z = -7.79, p = 0.00 | | | | | | | |
| **prospective** | | | | | | | |
| Rossetti 2024 | 12 | 86.67 | 11.26 | 12 | 100.2 | 12.47 | -13.53 [ -23.04, -4.02] |
| Paoli 2020 | 14 | 96.69 | 3.82 | 14 | 100.7 | 4.81 | -4.01 [ -7.23, -0.79] |
| Cincione 2021 | 17 | 88.94 | 10.72 | 17 | 98.38 | 10.45 | -9.44 [ -16.56, -2.32] |
| Heterogeneity: $\tau^2$ = 13.83, $I^2$ = 57.12%, $H^2$ = 2.33 | | | | | | | -7.68 [ -13.24, -2.12] |
| Test of $\theta_i = \theta_j$: Q(2) = 4.73, p = 0.09 | | | | | | | |
| Test of θ = 0: z = -2.71, p = 0.01 | | | | | | | |
| **retrospective** | | | | | | | |
| Magagnini 2022 | 25 | 80 | 3.2 | 25 | 88.4 | 3.2 | -8.40 [ -10.17, -6.63] |
| Heterogeneity: $\tau^2$ = 0.00, $I^2$ = .%, $H^2$ = . | | | | | | | -8.40 [ -10.17, -6.63] |
| Test of $\theta_i = \theta_j$: Q(0) = 0.00, p = . | | | | | | | |
| Test of θ = 0: z = -9.28, p = 0.00 | | | | | | | |
| **Overall** | | | | | | | -8.42 [ -10.72, -6.11] |
| Heterogeneity: $\tau^2$ = 4.41, $I^2$ = 53.16%, $H^2$ = 2.13 | | | | | | | |
| Test of $\theta_i = \theta_j$: Q(6) = 11.50, p = 0.07 | | | | | | | |
| Test of θ = 0: z = -7.15, p = 0.00 | | | | | | | |
| Test of group differences: $Q_b(2)$ = 1.21, p = 0.55 | | | | | | | |

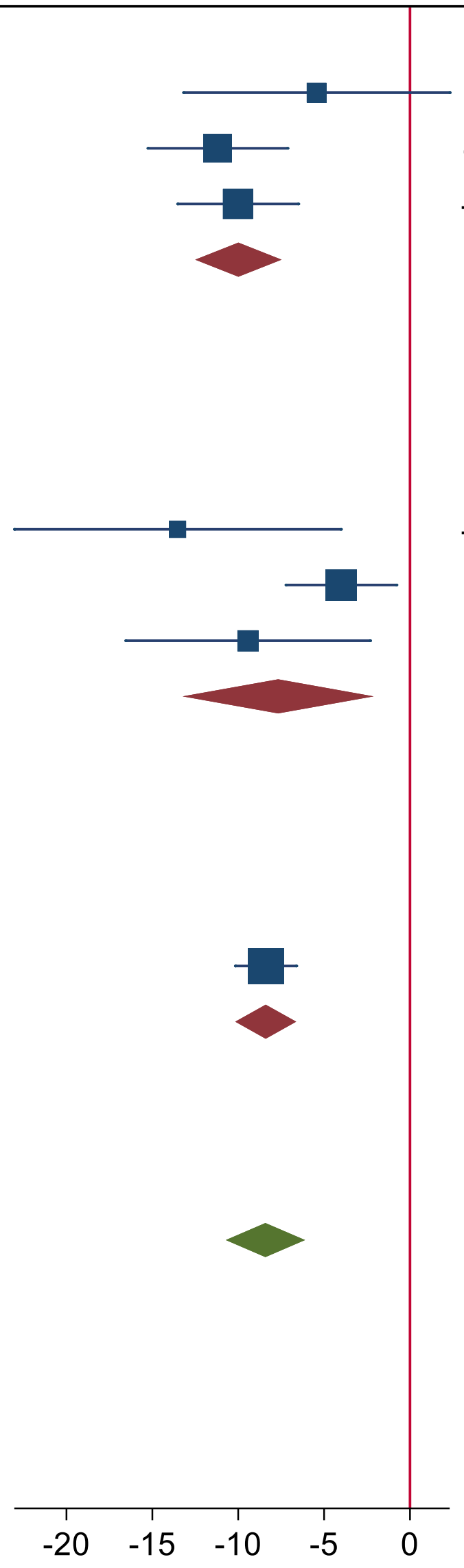


Random-effects REML model
Sorted by: sample
