# Supplementary material for: Ketogenic diet as a therapeutic intervention for biochemical hyperandrogenism in PCOS women with obesity or overweight: a meta-analytic study
Source: J Endocrinol Invest. 2026 Jun 13;49(11):3185–202. doi: 10.1007/s40618-026-02924-1 (PMC13722772; doi:10.1007/s40618-026-02924-1)
Supplement: Supplementary file 8 [file 40618_2026_2924_MOESM6_ESM.pdf]

WHR

Study	Treatment N	Treatment Mean	Treatment SD	Control N	Control Mean	Control SD	Mean diff. with 95% CI
**RCT**							
Li 2021	8	0.88	0.04	8	0.93	0.05	-0.05 [-0.09, -0.01]
Sharifi 2024	19	0.91	0.05	19	0.92	0.03	-0.01 [-0.03, 0.02]
Cincione 2023	73	0.78	0.06	73	0.81	0.06	-0.03 [-0.05, -0.01]
Heterogeneity: $\tau^2 = 0.00$, $I^2 = 44.15\%$, $H^2 = 1.79$							-0.02 [-0.05, -0.00]
Test of $\theta_i = \theta_j$: Q(2) = 3.67, p = 0.16							
Test of $\theta = 0$: z = -2.28, p = 0.02							
**prospective**							
Rossetti 2024	12	0.82	0.05	12	0.87	0.05	-0.05 [-0.09, -0.01]
Cincione 2021	17	0.80	0.03	17	0.82	0.02	-0.02 [-0.04, -0.00]
Meneghini 2023	42	0.85	0.07	42	0.88	0.14	-0.03 [-0.08, 0.02]
Heterogeneity: $\tau^2 = 0.00$, $I^2 = 12.30\%$, $H^2 = 1.14$							-0.03 [-0.04, -0.01]
Test of $\theta_i = \theta_j$: Q(2) = 1.87, p = 0.39							
Test of $\theta = 0$: z = -2.95, p = 0.00							
**Overall**							-0.02 [-0.03, -0.01]
Heterogeneity: $\tau^2 = 0.00$, $I^2 = 0.00\%$, $H^2 = 1.00$							
Test of $\theta_i = \theta_j$: Q(5) = 5.56, p = 0.35							
Test of $\theta = 0$: z = -4.60, p = 0.00							
Test of group differences: $Q_b(1) = 0.02$, p = 0.89							

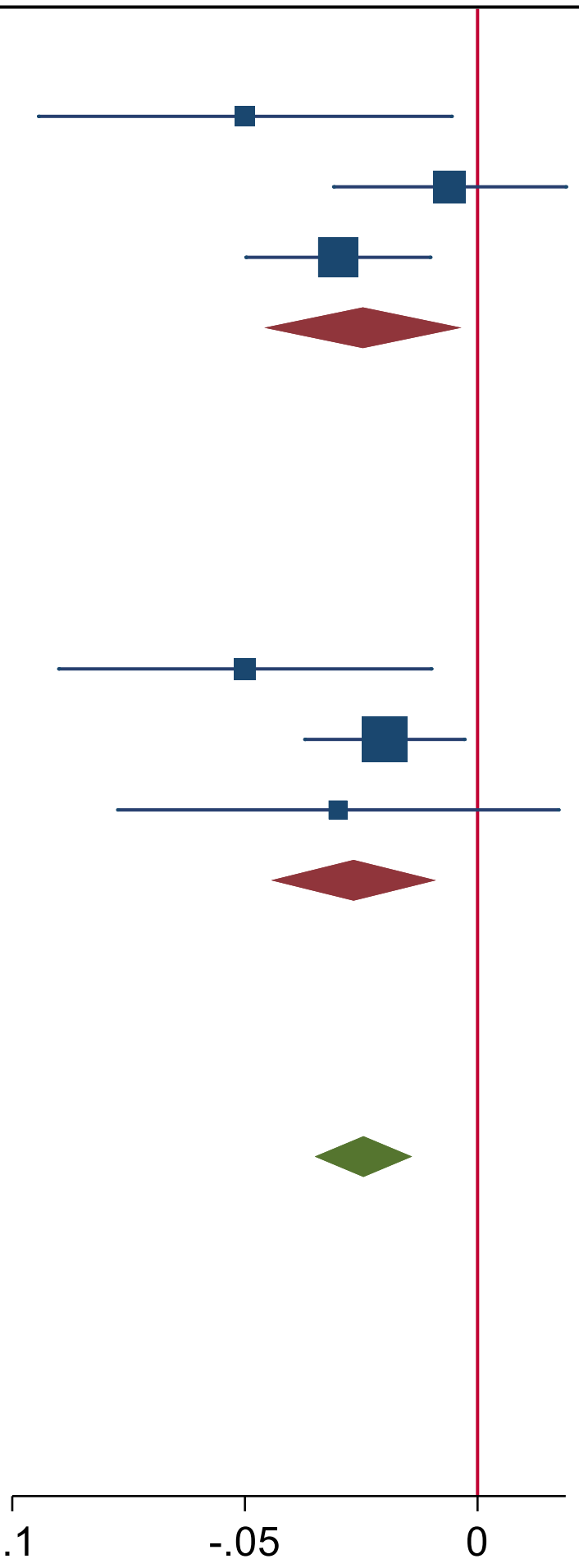

Random-effects REML model
Sorted by: sample
