# Supplementary material for: Ketogenic diet as a therapeutic intervention for biochemical hyperandrogenism in PCOS women with obesity or overweight: a meta-analytic study
Source: J Endocrinol Invest. 2026 Jun 13;49(11):3185–202. doi: 10.1007/s40618-026-02924-1 (PMC13722772; doi:10.1007/s40618-026-02924-1)
Supplement: Supplementary file 9 [file 40618_2026_2924_MOESM7_ESM.pdf]

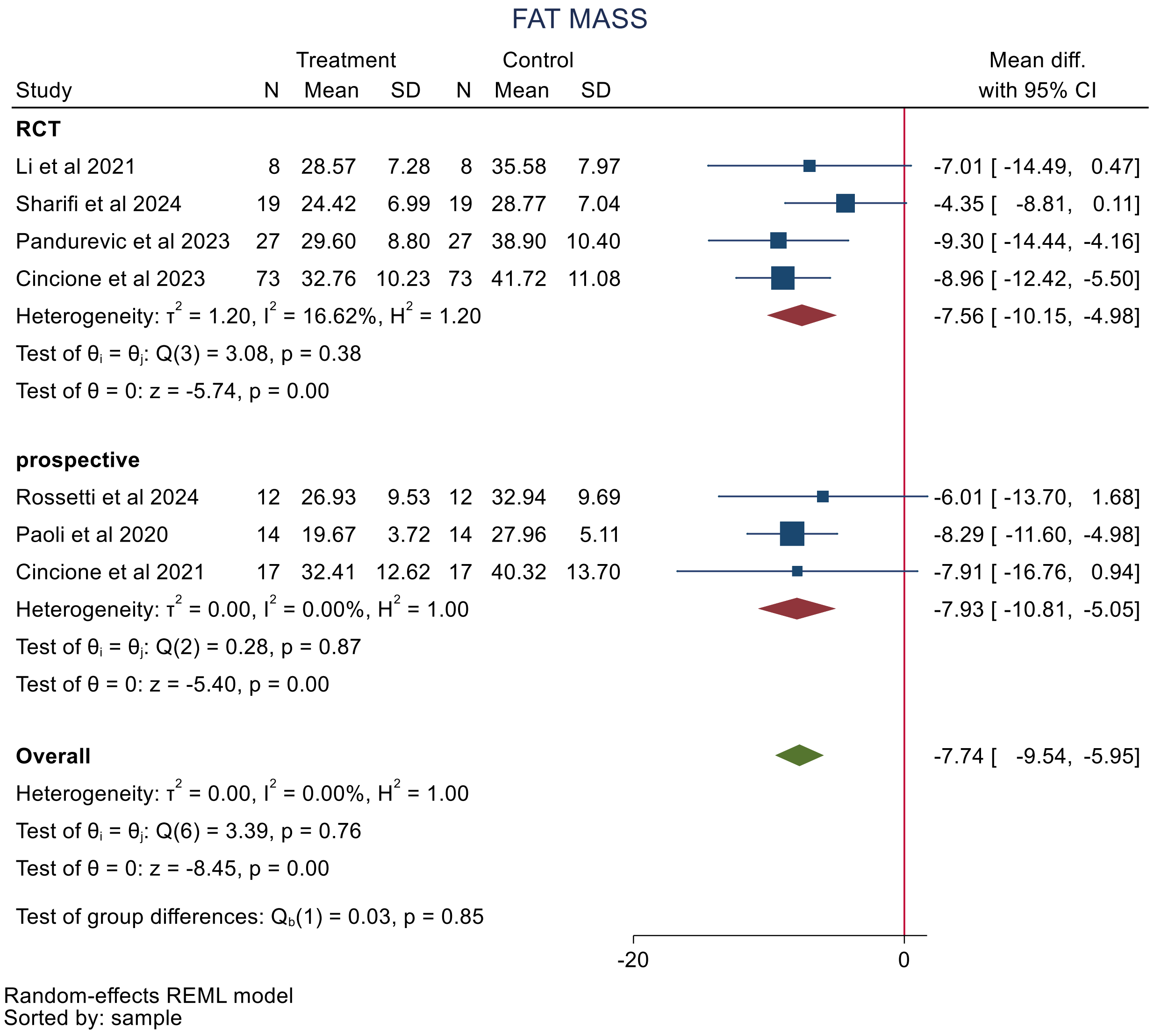
FAT MASS
Treatment
Control
Mean diff.
with 95% CI
Study
N
Mean
SD
N
Mean
SD
RCT
Li et al 2021 8 28.57 7.28 8 35.58 7.97 -7.01 [-14.49, 0.47]
Sharifi et al 2024 19 24.42 6.99 19 28.77 7.04 -4.35 [-8.81, 0.11]
Pandurevic et al 2023 27 29.60 8.80 27 38.90 10.40 -9.30 [-14.44, -4.16]
Cincione et al 2023 73 32.76 10.23 73 41.72 11.08 -8.96 [-12.42, -5.50]
Heterogeneity: τ² = 1.20, I² = 16.62%, H² = 1.20 -7.56 [-10.15, -4.98]
Test of θi = θj: Q(3) = 3.08, p = 0.38
Test of θ = 0: z = -5.74, p = 0.00
prospective
Rossetti et al 2024 12 26.93 9.53 12 32.94 9.69 -6.01 [-13.70, 1.68]
Paoli et al 2020 14 19.67 3.72 14 27.96 5.11 -8.29 [-11.60, -4.98]
Cincione et al 2021 17 32.41 12.62 17 40.32 13.70 -7.91 [-16.76, 0.94]
Heterogeneity: τ² = 0.00, I² = 0.00%, H² = 1.00 -7.93 [-10.81, -5.05]
Test of θi = θj: Q(2) = 0.28, p = 0.87
Test of θ = 0: z = -5.40, p = 0.00
Overall -7.74 [-9.54, -5.95]
Heterogeneity: τ² = 0.00, I² = 0.00%, H² = 1.00
Test of θi = θj: Q(6) = 3.39, p = 0.76
Test of θ = 0: z = -8.45, p = 0.00
Test of group differences: Qb(1) = 0.03, p = 0.85
-20
0
Random-effects REML model
Sorted by: sample
