# Supplementary material for: Ketogenic diet as a therapeutic intervention for biochemical hyperandrogenism in PCOS women with obesity or overweight: a meta-analytic study
Source: J Endocrinol Invest. 2026 Jun 13;49(11):3185–202. doi: 10.1007/s40618-026-02924-1 (PMC13722772; doi:10.1007/s40618-026-02924-1)
Supplement: Supplementary file 10 [file 40618_2026_2924_MOESM8_ESM.pdf]

FG

Study	Treatment N	Treatment Mean	Treatment SD	Control N	Control Mean	Control SD	Mean diff. with 95% CI
**RCT**							
Li 2021	8	92.00	8.30	8	106.60	15.30	-14.60 [-26.66, -2.54]
Sharifi 2024	19	87.94	2.67	19	96.21	4.68	-8.27 [-10.69, -5.85]
Pandurevic 2023	27	84.90	7.90	27	82.20	6.70	2.70 [-1.21, 6.61]
Cincione 2023	73	82.44	6.66	73	95.67	7.99	-13.23 [-15.62, -10.84]
Heterogeneity: $\tau^2 = 54.44$, $I^2 = 94.86\%$, $H^2 = 19.47$							-7.79 [-15.56, -0.03]
Test of $\theta_i = \theta_j$: Q(3) = 47.59, p = 0.00							
Test of θ = 0: z = -1.97, p = 0.05							
**prospective**							
Rossetti 2024	12	82.00	13.85	12	84.08	15.34	-2.08 [-13.77, 9.61]
Paoli 2020	14	83.59	4.32	14	91.88	4.50	-8.29 [-11.56, -5.02]
Cincione 2021	17	85.14	8.17	17	95.21	8.59	-10.07 [-15.71, -4.43]
Yang 2022	55	90.01	22.79	55	96.74	22.79	-6.73 [-15.25, 1.79]
Heterogeneity: $\tau^2 = 0.00$, $I^2 = 0.00\%$, $H^2 = 1.00$							-8.22 [-10.83, -5.60]
Test of $\theta_i = \theta_j$: Q(3) = 1.59, p = 0.66							
Test of θ = 0: z = -6.16, p = 0.00							
**retrospective**							
Meng 2025	35	81.07	8.11	35	90.79	10.63	-9.72 [-14.15, -5.29]
Heterogeneity: $\tau^2 = 0.00$, $I^2 = .\%$, $H^2 = .$							-9.72 [-14.15, -5.29]
Test of $\theta_i = \theta_j$: Q(0) = 0.00, p = .							
Test of θ = 0: z = -4.30, p = 0.00							
**Overall**							-7.80 [-11.39, -4.21]
Heterogeneity: $\tau^2 = 21.49$, $I^2 = 83.51\%$, $H^2 = 6.06$							
Test of $\theta_i = \theta_j$: Q(8) = 49.52, p = 0.00							
Test of θ = 0: z = -4.26, p = 0.00							
Test of group differences: $Q_b(2) = 0.37$, p = 0.83							

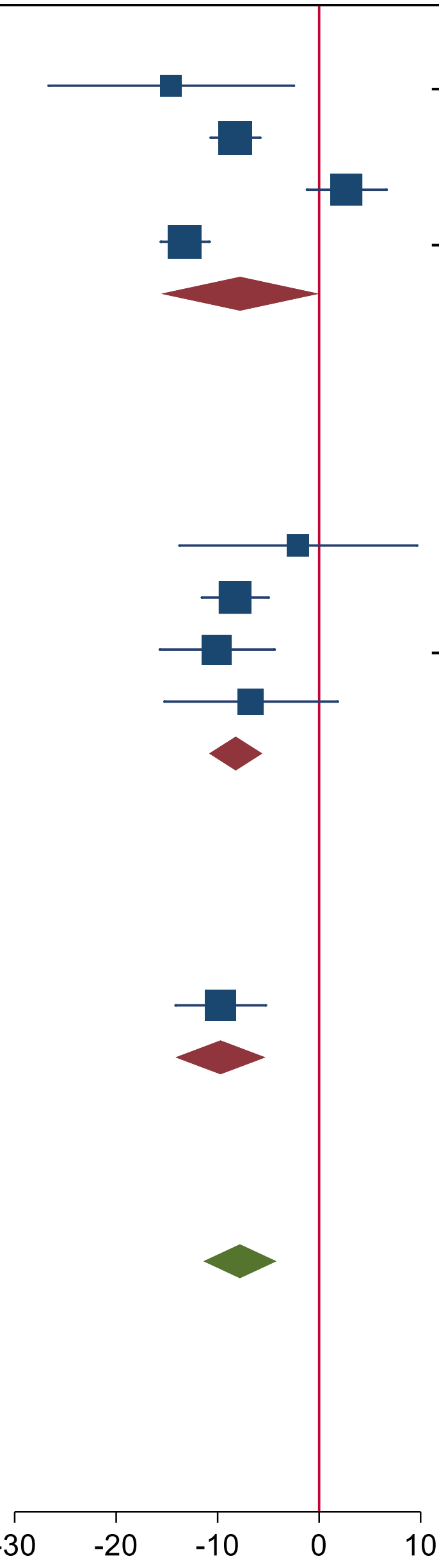

Random-effects REML model
Sorted by: sample
