# Supplementary material for: Ketogenic diet as a therapeutic intervention for biochemical hyperandrogenism in PCOS women with obesity or overweight: a meta-analytic study
Source: J Endocrinol Invest. 2026 Jun 13;49(11):3185–202. doi: 10.1007/s40618-026-02924-1 (PMC13722772; doi:10.1007/s40618-026-02924-1)
Supplement: Supplementary file 11 [file 40618_2026_2924_MOESM9_ESM.pdf]

FI

Study	Treatment N	Treatment Mean	Treatment SD	Control N	Control Mean	Control SD	Mean diff. with 95% CI
**RCT**							
Sharifi 2024	19	14.45	3.10	19	27.57	3.17	-13.12 [-15.11, -11.13]
Pandurevic 2023	27	9.90	5.60	27	13.40	7.00	-3.50 [-6.88, -0.12]
Cincione 2023	73	16.29	7.40	73	37.93	20.71	-21.64 [-26.68, -16.60]
Heterogeneity: $\tau^2 = 76.46$, $I^2 = 96.48\%$, $H^2 = 28.43$							-12.63 [-22.74, -2.51]
Test of $\theta_i = \theta_j$: Q(2) = 39.44, p = 0.00							
Test of $\theta = 0$: z = -2.45, p = 0.01							
**prospective**							
Rossetti 2024	12	16.03	15.59	12	25.72	14.06	-9.69 [-21.57, 2.19]
Paoli 2020	14	11.31	0.60	14	12.62	0.48	-1.31 [-1.71, -0.91]
Cincione 2021	17	11.95	7.59	17	24.85	22.18	-12.90 [-24.04, -1.76]
Heterogeneity: $\tau^2 = 30.86$, $I^2 = 64.18\%$, $H^2 = 2.79$							-6.19 [-14.00, 1.62]
Test of $\theta_i = \theta_j$: Q(2) = 6.05, p = 0.05							
Test of $\theta = 0$: z = -1.55, p = 0.12							
**retrospective**							
Meng 2025	35	13.71	4.48	35	18.09	7.46	-4.38 [-7.26, -1.50]
Heterogeneity: $\tau^2 = 0.00$, $I^2 = .\%$, $H^2 = .$							-4.38 [-7.26, -1.50]
Test of $\theta_i = \theta_j$: Q(0) = 0.00, p = .							
Test of $\theta = 0$: z = -2.98, p = 0.00							
**Overall**							-9.11 [-14.79, -3.43]
Heterogeneity: $\tau^2 = 49.86$, $I^2 = 96.89\%$, $H^2 = 32.13$							
Test of $\theta_i = \theta_j$: Q(6) = 197.67, p = 0.00							
Test of $\theta = 0$: z = -3.15, p = 0.00							
Test of group differences: $Q_b(2) = 2.44$, p = 0.30							

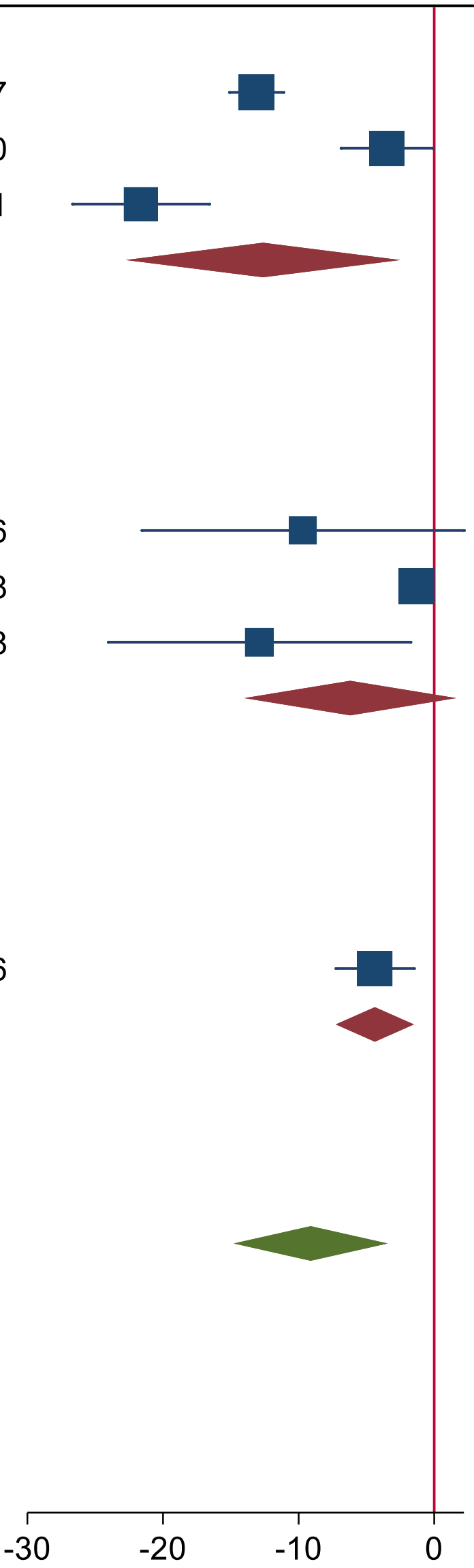

Random-effects REML model
Sorted by: sample
