# Supplementary material for: Ketogenic diet as a therapeutic intervention for biochemical hyperandrogenism in PCOS women with obesity or overweight: a meta-analytic study
Source: J Endocrinol Invest. 2026 Jun 13;49(11):3185–202. doi: 10.1007/s40618-026-02924-1 (PMC13722772; doi:10.1007/s40618-026-02924-1)
Supplement: Supplementary file 12 [file 40618_2026_2924_MOESM1_ESM.docx]

**Supplementary Figure 1**. Mean Differences with 95% Confidence Interval (CI) in BMI (Body Mass Index) in Patients with Polycystic Ovary Syndrome Undergoing a Ketogenic Diet Protocol. The analysis includes prospective and retrospective studies and controlled clinical trials (RCT).

**Supplementary Figure 2**. Mean Differences with 95% Confidence Interval (CI) in WC (Waist circumference) in Patients with Polycystic Ovary Syndrome Undergoing a Ketogenic Diet Protocol. The analysis includes prospective and retrospective studies and controlled clinical trials (RCT).

**Supplementary Figure 3**. Mean Differences with 95% Confidence Interval (CI) in WHR (waist-to-hip ratio) in Patients with Polycystic Ovary Syndrome Undergoing a Ketogenic Diet Protocol. The analysis includes prospective and retrospective studies and controlled clinical trials (RCT).

**Supplementary Figure 4**. Mean Differences with 95% Confidence Interval (CI) in fat mass in Patients with Polycystic Ovary Syndrome Undergoing a Ketogenic Diet Protocol. The analysis includes prospective and retrospective studies and controlled clinical trials (RCT).

**Supplementary Figure 5**. Mean Differences with 95% Confidence Interval (CI) in fasting glucose levels in Patients with Polycystic Ovary Syndrome Undergoing a Ketogenic Diet Protocol. The analysis includes prospective and retrospective studies and controlled clinical trials (RCT).

**Supplementary Figure 6**. Mean Differences with 95% Confidence Interval (CI) in fasting insulin levels in Patients with Polycystic Ovary Syndrome Undergoing a Ketogenic Diet Protocol. The analysis includes prospective and retrospective studies and controlled clinical trials (RCT).

**Supplementary Figure 7**. Mean Differences with 95% Confidence Interval (CI) in HOMA-IR (Homeostatic Model Assessment for Insulin Resistance) levels in Patients with Polycystic Ovary Syndrome Undergoing a Ketogenic Diet Protocol. The analysis includes prospective and retrospective studies and controlled clinical trials (RCT).

**Supplementary Figure 8**. Mean Differences with 95% Confidence Interval (CI) in Total cholesterol levels in Patients with Polycystic Ovary Syndrome Undergoing a Ketogenic Diet Protocol. The analysis includes prospective and retrospective studies and controlled clinical trials (RCT).

**Supplementary Figure 9**. Mean Differences with 95% Confidence Interval (CI) in Triglycerides levels in Patients with Polycystic Ovary Syndrome Undergoing a Ketogenic Diet Protocol. The analysis includes prospective and retrospective studies and controlled clinical trials (RCT).

**Supplementary Figure 10**. Mean Differences with 95% Confidence Interval (CI) in HDL (high-density lipoprotein) cholesterol levels in Patients with Polycystic Ovary Syndrome Undergoing a Ketogenic Diet Protocol. The analysis includes prospective and retrospective studies and controlled clinical trials (RCT).

**Supplementary Figure 11**. Mean Differences with 95% Confidence Interval (CI) in LDL (low-density lipoprotein) cholesterol levels in Patients with Polycystic Ovary Syndrome Undergoing a Ketogenic Diet Protocol. The analysis includes prospective and retrospective studies and controlled clinical trials (RCT).
